# Leaf: an ultrafast filter for population-scale long-read SV detection

**DOI:** 10.1186/s13059-024-03297-5

**Published:** 2024-06-13

**Authors:** Chenxu Pan, Knut Reinert

**Affiliations:** 1 Department of Mathematics and Computer Science, Freie Universität Berlin, Takustr. 9, 14195 Berlin, Germany; 2 Department of Computational Molecular Biology, Max Planck Institute for Molecular Genetics, Berlin, 14195 Germany

**Keywords:** Filter-based pipelines, Intra-read SV detection, Population-scale long-read applications, Generative model, Extended SAM/BAM

## Abstract

Advances in sequencing technology have facilitated population-scale long-read structural variant (SV) detection. Arguably, one of the main challenges in population-scale analysis is developing effective computational pipelines. Here, we present a new filter-based pipeline for population-scale long-read SV detection. It better captures SV signals at an early stage than conventional assembly-based or alignment-based pipelines. Assessments in this work suggest that the filter-based pipeline helps better resolve intra-read rearrangements. Moreover, it is also more computationally efficient than conventional pipelines and thus may facilitate population-scale long-read applications.

**Supplementary Information:**

The online version contains supplementary material available at 10.1186/s13059-024-03297-5.

## Background

Advancements in long-read sequencing have reached a level of accuracy and yield that allows population-scale applications [1]. Pacific Biosciences (PacBio) and Oxford Nanopore Technologies (ONT) are the two leading long-read sequencing platforms in the field. The PacBio platform can generate high fidelity (HiFi) reads, which are > 15 Kbps highly accurate reads [2]. The ONT platform can produce much longer reads (> 4 Mbps) at a lower cost, while the reads are less accurate [3]. Existing research has shown that long-read sequencing can discover a substantial proportion of previously undetected SVs [4–10]. Long-read sequencing research in recent years has provided insight into structural variants at a population level, such as the study of structural variants in the sequencing of 3622 Icelanders [11] and the Human Pangenome Project [12], which creates a more sophisticated and complete human reference genome of global genomic diversity. Long-read sequencing has also been applied to population-scale SV detection in fields like agriculture [14–15] and metagenomics [16, 17].

Ongoing advances in computational tools in the past years have facilitated long-read applications [18–20]. Alignment and de novo assembly are the main approaches for long-read sequencing analysis 21 [22–25]. Assembly-based approaches are commonly more effective in reconstructing highly diverse structures in sequences than alignment-based approaches [26, 27]. Nevertheless, de novo assembly requires higher read coverage and more computationally demanding [28], and thus it is challenging to apply assembly-based approaches to population-scale sequencing analysis [13, 14, 29, 30]. Population-scale analytical pipelines are supposed to be both effective and efficient [31, 32]. Although more advanced tools are constantly introduced in the rapidly developing areas [33–37]. Arguably, the main challenge in population-scale applications remains developing efficient and scalable analytical pipelines.

Here, we propose the filter-based pipeline for population-scale long-read SV detection. Different from conventional pipelines, such as assembly- or alignment-based ones, filter-based pipelines capture SV signals at a very early stage. Intuitively, it would be helpful to detect SV signals at an early stage because of the ultra-long read potentially containing intra-reads SVs that are likely missed by many existing assembly or alignment-based methods. To validate the feasibility of filter-based pipelines, we implemented Leaf (i.e., LinEAr Filter) within our long-read computational toolkit Linear. Assessments based on high-quality datasets and benchmark tools in this work suggest that filter-based pipelines are comparable to or outperform conventional pipelines in terms of detecting complex intra-read rearrangements and computational efficiency.

## Results

**Fig. 1:**
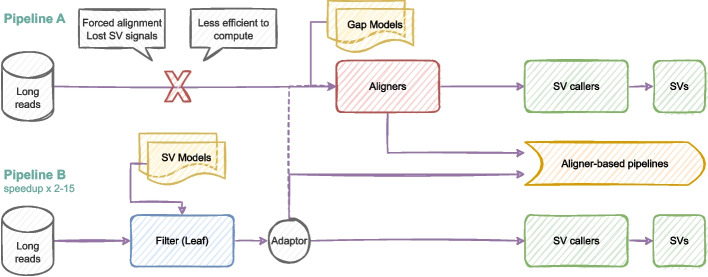
The aligner-based long-red SV detection pipeline (**A**) compared to the filter-based pipeline (**B**), which applies SV models at an early stage to better capture long-read SV signals

Aligner-based long-read SV detection pipelines, as shown in pipeline A Fig. 1, rely on SV callers to resolve intra-read SVs. Commonly, long-read aligners are capable of mapping intra-read insertions and deletions by employing nonlinear models (e.g., convex model) at the cost of largely increased computational complexity. However, aligners remain less effective in mapping more complex intra-read SVs, especially nonlinear ones (e.g., inverted, duplicated and nested). The underlying cause is the alignment algorithm complexity that limits the capability of thoroughly taking into account potential rearrangements. The limitation may less impact short-read SV detection since most of them are inter-read SVs supposed to be detected by SV callers. However, due to the ultra-long lengths, long reads commonly contain a significantly larger number of intra-read SVs, which can hardly be detected by SV callers if the alignment loses the SV signals by, for instance, forced alignment. Therefore, a lightweight approach that can capture SV signals at an early stage would be helpful for long-read SV detection. To this end, we propose the filter-based pipeline as shown in pipeline B Fig. 1.

In the following sections, we will discuss the assessment of Leaf-based pipelines based on three high-quality datasets and benchmark tools, which include:

1. Trio-based assessments based on the Mendelian inheritance.
2. Systematical simulation of intra-read SVs for evaluating the detectable SV space.
3. Assembly-based SV calls for HiFi read insertion and deletion detection evaluation.

### Trio-based SV call assessment

We prepared 7 datasets for the trio-based assessment:

1. Ashkenazim Jewish trio: HG002 (son), HG003 (father), and HG004 (mother) [37, 38];
2. Han Chinese trio: HG005 (son), HG006 (father), and HG007 (mother) [37]; and
3. SKBR3 breast cancer cell line [39].

**Fig. 2:**
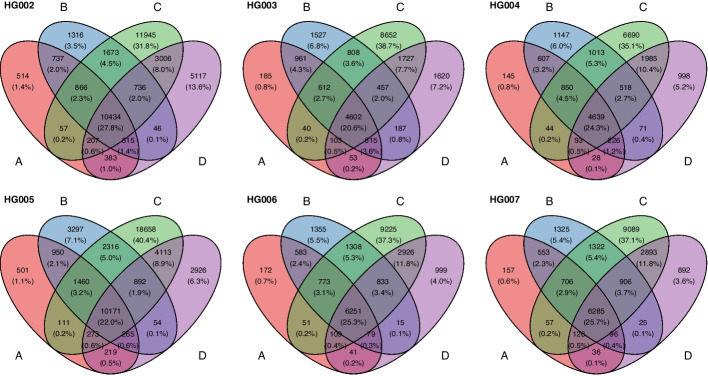
Venn diagrams of SVs detected by 4 different pipelines (**A**–**D**) on 6 datasets of 2 trios, Ashkenazim Jewish trio (HG002–HG004) and Han Chinese trio (HG005–HG007). Pipeline A uses the aligner and SVIM. Pipeline B uses the aligner and cuteSV. Pipeline C uses Leaf and cuteSV. Pipeline D uses Leaf and SVIM

We set up 4 different pipelines combining Leaf, long-read aligner minimap2 [22] with two SV callers, SVIM [40] and cuteSV [41], to call SVs in the datasets described above. First, we set the minimum number of reads to call an SV (supporting reads) 7 to get an overview of the number of SVs detected by each pipeline. Figure 2 compares the number of SVs detected by the four pipelines. Table 1 summarizes SVs ≥ 80 bps detected by the Leaf-cuteSV and Leaf-SVIM. We employ the relative recall [43] in the following expression to compare the number of SVs of the *i*th dataset detected by pipelines *X* and $Y\in \{A,B,C,D\}$.

1 $$\begin{aligned} {recall}_{X_i|Y_i}=\dfrac{|X_i\cap Y_i|}{|Y_i|}=\dfrac{{ SVs\ in\ }X_i{\ and\ } Y_i}{{\ SVs\ in }\ Y_i} \end{aligned}$$

For instance, $recall_{C_2\vert A_2}=0.843$ means that for HG002, pipeline *C* (Leaf-cuteSV) recalls $84.3\%$ SVs detected by pipeline *A* (aligner-SVIM). The average relative recall over all datasets for each pipeline ($recall_{C\vert A}=0.848$, $recall_{A\vert C}=0.657$, $recall_{C\vert B}=0.794$ and $recall_{B\vert C}=0.576$) suggests that Leaf-based pipelines (*C* and *D*) recall more SVs than aligner-based pipelines (*A* and *B*).

**Table 1:** Summary of SVs (≥ 80 bps) detected by Leaf-SV callers ( ≥ 80 supporting reads)

Trios	Dataset	Platform	Depth	SVs caller	Total	INS	DEL	INV	DUP
Ashkenazim	HG002 (son)	PacBio	72	cuteSV	28,924	13,261	11,819	460	3384
Jewish	HG002 (son)	PacBio	72	SVIM	20,444	9742	9943	/	759
	HG002 (son)	ONT	50	cuteSV	25,258	11,068	10,821	113	3256
	HG002 (son)	ONT	50	SVIM	17,774	7789	9985	/	/
	HG003 (father)	PacBio	32	cuteSV	17,001	7974	8006	197	824
	HG003 (father)	PacBio	32	SVIM	9564	4462	4959	/	143
	HG004 (mother)	PacBio	32	cuteSV	15,832	7155	8001	78	598
	HG004 (mother)	PacBio	32	SVIM	8558	4076	4385	/	97
Han Chinese	HG005 (son)	PacBio	63	cuteSV	37,994	21,244	13,436	431	2883
	HG005 (son)	PacBio	63	SVIM	18,913	8676	9689	/	548
	HG006 (father)	PacBio	30	cuteSV	21,476	10,215	10,193	189	879
	HG006 (father)	PacBio	30	SVIM	11,253	5414	5693	/	146
	HG007 (mother)	PacBio	30	cuteSV	21,384	10,409	9790	162	1023
	HG007 (mother)	PacBio	30	SVIM	11,259	5487	5649	/	123
/	SKBR3 cell line	PacBio	72	cuteSV	34,436	18,423	12,312	206	3495
	SKBR3 cell line	PacBio	72	SVIM	19,381	9141	9414	/	826

We then employ the Mendelian inheritance [23, 41, 43] to evaluate the recall and precision of each pipeline. We preparedto evaluate the recall and precision of each pipeline. We prepared the high-confidence SV datasets denoted as *T* for both the Ashkenazim Jewish trios and Han Chinese trios. We established the criteria that each high-confidence SV $\in T$ must be recalled by an SV caller with a minimum of 10 supporting reads and must align with Mendelian inheritance. The comparison of two SVs involves assessing their reciprocal overlap, deeming them identical if a proportion of their individual sizes overlap, and their genotypes match. For insertions and duplications, which lack a physical span over the reference, we compare their virtual reference span defined as a span starting at the SV position and ending at the virtual endpoint an SV length away from the starting endpoint. We employed the true positive rate (TPR), Mendelian discordance rate (MDR), and recall of homozygous (RH) given by the following expressions for evaluation,

$$\begin{aligned} TPR=recall_{{son}|{T}}=\frac{{son's\ true\ SVs }}{{true\ SVs}} \end{aligned}$$

$$\begin{aligned} {MDR}={recall}_{\overline{{parents}}|{son}}=\dfrac{{son's\ SVs\ not\ detected\ in\ parents}}{{son's\ SVs}} \end{aligned}$$

$$\begin{aligned} {RH}={recall}_{{son}|{parents\ homozygous}}=\dfrac{{parents\ homozygous\ SVs\ detected\ in\ son}}{{parents\ homozygous\ SVs}} \end{aligned}$$

where $recall_{X|Y}$ is the relative recall defined in expression Eq. 1.

Table 2 summarizes TPRs, MDRs, and RHs of the four pipelines applied to the two trios. The results suggest aligner-based pipelines have relatively better MDRs and RHs, while Leaf-based pipelines have much better TPRs. MDRs ($>10\%$) of both types of pipelines are significant, particularly when combined with cuteSV, revealing that some SVs detected in sons do not follow Mendelian inheritance. However, they are largely attributable to the lower read coverage of parents (30×) compared to sons (72×). Moreover, it is also worth noting that the two SV callers perform differently in terms of recall and precision. SVIM generates fewer false positives (lower MDRs and higher RHs), while cuteSV reports more true SVs (higher TPRs). The statistics suggest SVs recalled by the four pipelines are basically in line with the Mendelian inheritance, while Leaf-SV callers reported more SVs that passed the Mendelian inheritance validation.

**Table 2:** True positive rate (TPR), Mendelian discordance rate (MDR) and recall of homozygous (RH) for the two trios. Highlighted numbers are better

Trios	SVs caller	TPR[%]		MDR[%]		RH[%]	
		Leaf	Aligner	Leaf	Aligner	Leaf	Aligner
Ashkenazim Jewish	cuteSV	**93.21**	67.98	24.40	**22.16**	89.13	**95.46**
	SVIM	**63.10**	57.04	18.70	**8.88**	**99.02**	98.07
Han Chinese	cuteSV	**88.88**	70.04	**18.90**	21.90	93.61	**96.23**
	SVIM	55.61	**56.67**	9.67	**6.79**	97.57	**97.70**

Read coverage is critical to population-scale long-read SV analysis due to the sequencing cost. Hence we assessed the precision, recall, and $F_1$ score (*F*-measure) as shown in the following expression corresponding to the number of supporting reads for calling SVs.

$$\begin{aligned} F_1=2\cdot \dfrac{{recall}\cdot {precision}}{{recall}+{precision}} \end{aligned}$$

**Fig. 3:**
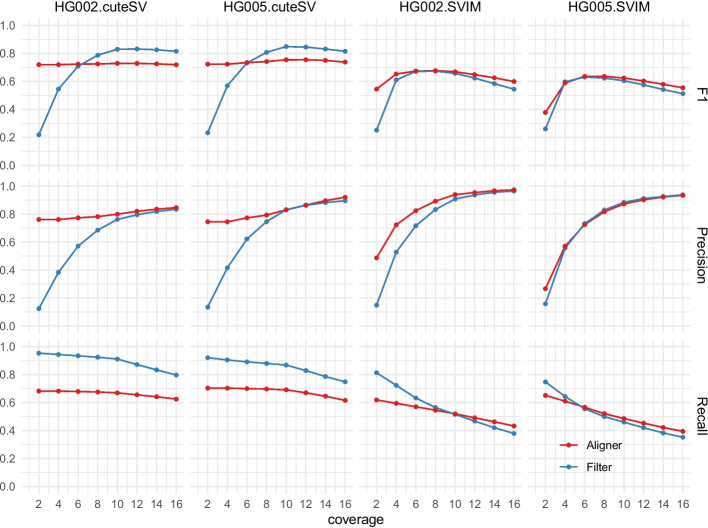
$F_1$ score, precision and recall for 4 pipelines across datasets of two sons (i.e., HG002 and HG005). The horizontal axis of coverage is the minimum number of supporting reads to recall an SV

The results are shown in Fig. 3, where the axis of coverage is the minimum supporting reads to recall an SV. It shows SVIM-based pipelines are of similar performances, while the cuteSV-based pipelines exhibit notable differences. Leaf-cuteSV has the highest recall at all levels of coverage. Its precision is lower than aligner-cuteSV, especially when coverage < 7, while the precision increases quickly and becomes comparable when coverage ≥ 8. Leaf-cuteSV with 8 to 10 supporting reads achieves the most balanced performance (i.e., highest $F_1$ score).

**Fig. 4:**
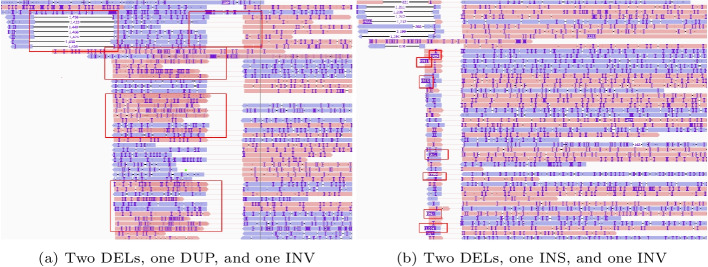
Highly nested SVs found in the SKBR3 breast cancer cell line by Leaf-cuteSV. Images are generated by Integrative Genomics Viewer (IGV). Sequences of different strands are highlighted with different colors. The nested SV in the first subfigure comprises two deletions of 1441 bps and 750 bps on the two sides and one 976-bps duplication in darker red embedded in the inversion. The nested SV in the second subfigure comprises two deletions and one 987-bps insertion highlighted by rectangles embedded in the inversion

Nested SVs are known to be associated with diseases, while SVs nested in long reads are commonly more difficult for aligner-based pipelines to resolve. We analyzed nested SVs comprising two basic SVs (i.e., INS, DEL, INV, DUP), such as inverted duplication (INVDUP), insertion nested inversion (INVINS), deletion nested inversion (INVDEL), and deletion nested duplication (DUPDEL), based on the results of Leaf- or aligner-SV caller pipelines. Table 3 summarizes the number of nested SVs found in the trio-based datasets and SKBR3 dataset. We did not assess the recall and precision due to lacking nested SV callers [18, 44]. Figure 4 shows two highly nested SVs comprising four basic ones in SKBR3 found by Leaf-cuteSV. It is to show the potential of filter-based pipelines in detecting highly nested SVs.

**Table 3:** Comparison of nested SVs found in the results of Leaf- and aligner(Aln)-cuteSV

Dataset	Total		INVDUP		INVINS		INVDEL		DUPDEL	
	Leaf	Aln	Leaf	Aln	Leaf	Aln	Leaf	Aln	Leaf	Aln
Ashkenazim Jewish son	127	66	17	3	7	4	7	9	96	50
Ashkenazim Jewish parents	91	29	18	1	4	3	3	3	66	22
Han Chinese son	189	85	21	3	9	10	9	14	150	58
Han Chinese parents	85	51	14	3	5	1	5	5	61	42
SKBR3	58	56	9	3	8	3	3	8	38	42

### Detectable SV space assessment

In this assessment, we systematically simulated intra-read SVs for measuring the detectable SV space of Leaf- and aligner-based pipelines. The long-read SV space in the assessment comprises three key attributes: SV type, SV length and sequencing error. We used long-read simulators PBSIM and NanoSim [45] to simulate PacBio and ONT reads sequenced from GRCH38 with the average sequencing errors of 10%, 15%, and 20%. SV types including insertion, deletion, duplication, and inversion of lengths ranging from 100 bps to $2\times10^3\;\mathrm{bps}$ are simulated and planted into simulated reads at random positions. We also employed two advanced long-read aligners, minimap2 and NGMLR [23] as the control. Then we ran Leaf and aligners and evaluated recall and precision by directly comparing the planted SV endpoint deviation, which is the distance between the detected SV endpoints and the planted ones, without using an SV caller since the planted SV endpoints are known. An SV is regarded as correctly identified if all endpoint deviations $\leq50\;\mathrm{bps}$.

**Fig. 5:**
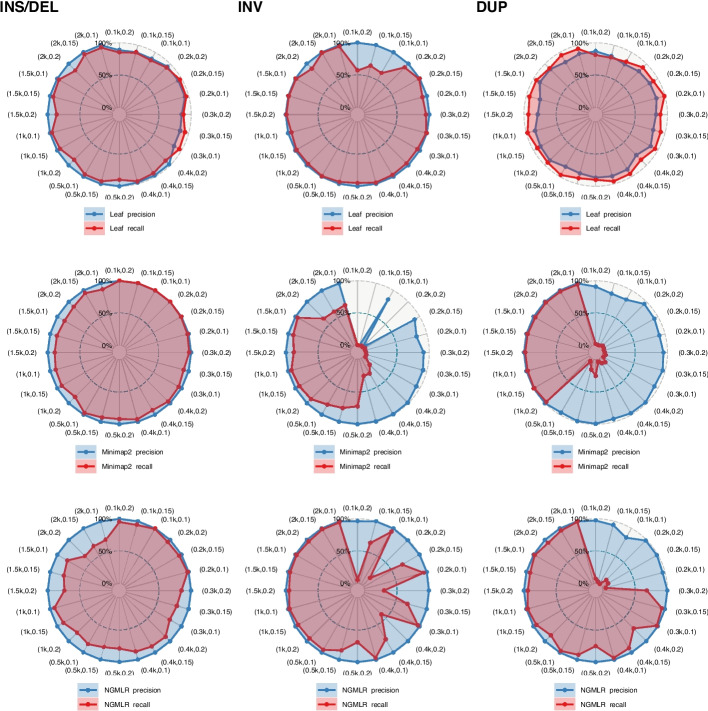
Detectable SV space of Leaf and aligners measured by the recall and precision of detecting systematically simulated SVs. Values in the figure are labeled by tuples of SV length (bps) and the sequencing error

Figure 5 shows the detectable SV space measured by recall and precision. As expected, aligners performed better in detecting insertions and deletions mostly because of the nonlinear gap model (e.g., convex gap model), which can distinguish between short indels of sequencing errors and longer insertions or deletions of SVs. However, the aligners are ineffective in mapping nonlinear intra-read SVs such as inversions and duplications as shown in the second and third rows of the figure. By contrast, Leaf is comparable to the aligners in detecting insertions and deletions, while it remains effective in mapping nonlinear SVs, such as inversions of 200 bps to 500 bps missed by aligners. Overall, Leaf shows more complete detectable SV space than aligners. The assessment suggests that canonical long-read pipelines, such as aligner-SV callers, could be substantially less effective in detecting nonlinear intra-read SVs. It is largely attributable to the incomplete space of aligners, which may lose critical SV signals, while the filter-based pipelines, which capture SV signals at an early stage, such as Leaf in the assessment, have the potential to complement the detectable SV space and thus enhance the capability of canonical pipelines in detecting complex SVs.

### Assembly-based SV call assessment

In this assessment, we evaluated the performance of Leaf-SV caller pipelines based on assembly-based insertion and deletion calls. The assessment workflow is shown in Additional File 1: Fig. S1. Specifically, we prepared an insertion and deletion dataset by applying pipeline dipcall [46] to the Human Pangenome Reference Consortium (HPRC) diploid assembly of HG00733 as the true SV set for evaluation. We then used the public dataset of HG00733 PacBio HiFi reads as the read datasets and applied Leaf, minimap2, and NGMLR with cuteSV and SVIM to the reads for SV calling. Finally, we compared SVs detected by the four pipelines to the assembly-based SV set by using the benchmark toolkit Truvari [43]. It is worth noting that the datasets of assessment are prepared based on minimap2. Specifically, dipcall is a pipeline employing minimap2 for aligning genome to HPRC assembly, which is also assembled by hifiasm developed by the team of minimap2. Additionally, both the HPRC assembly and the read dataset for testing are HG00733 PacBio HiFi reads. In such a case, the assessment essentially employs minimap2 as the benchmark for evaluating the precision and recall of other pipelines. Hence minimap2 in this test is employed as the reference bounds of recall and precision, and another different NGMLR-based pipeline is employed as the bias (confounder) control.

**Fig. 6:**
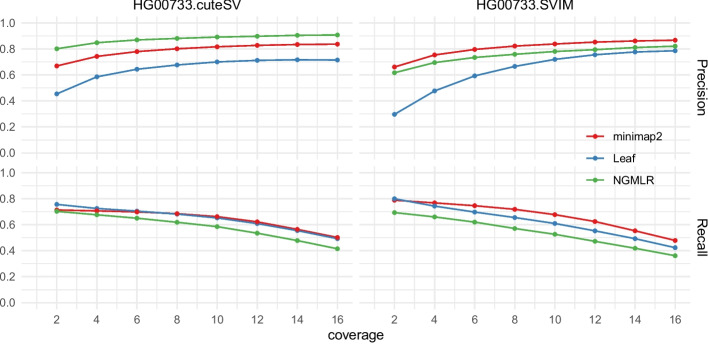
Results of detecting insertions and deletions in HG00733 for the six pipelines. The precision and recall are evaluated by comparing the results to the true SV set generated by minimap2-based pipelines (dipcall). The axis coverage is the minimum number of supporting reads to call an SV

Figure 6 shows the assessment results, where recall of Leaf is higher than that of NGMLR and is close to minimap2 in the assessment. Due to the assessment bias discussed above, the relative $recall_{Leaf|minimap2}$ defined in expression Eq. 1, is a better metric for recall assessment. Additional File 1: Table S1 summarizes the relative recall of Leaf-based pipelines. It suggests Leaf-SV callers can recall most insertions and deletions detected by minimap2-SV callers. On the other hand, the relatively lower precision of Leaf-based pipelines is attributable to that Leaf reported more SVs. However, it conforms to the general design principle of filters, where sensitivity commonly takes priority over others including precision, which can be easily improved in the validation stage. Moreover, a number of false positives are potential true SVs missed by dipcall. Existing research suggests this number could be upper to 15% [47]. Thus the real recall and precision of Leaf could be substantially higher.

### Computational performance assessment

**Fig. 7:**
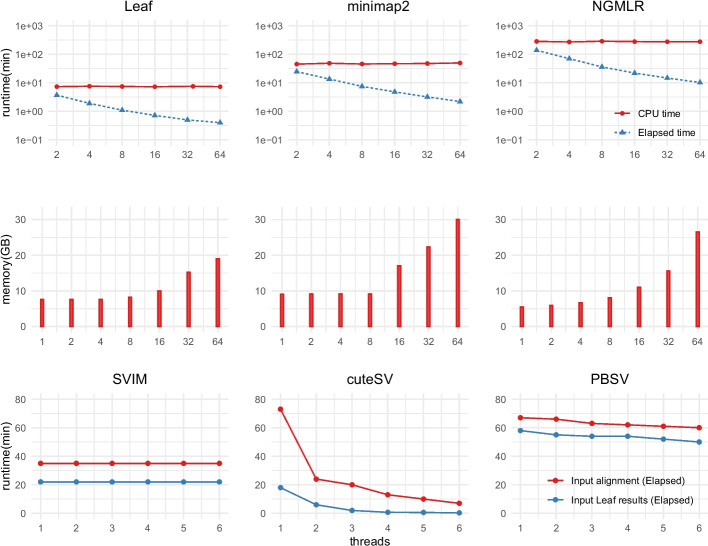
Runtime and memory footprint assessment: The first and second rows are the time and maximum resident memory of Leaf and aligners. CPU time is the amount of time for which the CPU is used. Elapsed time is the time for which the program runs. Vertical axes in the first row are in $\log _{10}$ scaled. The last row is the runtime of SV callers taking alignment and Leaf results of PacBio CLR reads as input

Finally, we assessed the computational performance of Leaf- and aligner-based (i.e., minimap2 and NGMLR) pipelines. Without loss of generality, we used PacBio raw reads of the HG002 dataset for the evaluation instead of HiFi reads since it is commonly more computationally intensive to process raw reads. We evaluated the runtime and memory footprint for running Leaf, minimap2 and NGMLR. Both aligners apply the single instruction multiple data (SIMD), which is a parallelism technique for hardware acceleration, to accelerate the gap model for insertion and deletion detection. Therefore, they run much faster than many other long-read aligners. We evaluated the elapsed time as well as the CPU time, which is a better metric for assessing algorithm complexity excluding I/O. In the results as shown in Fig. 7, Leaf runs significantly faster than the aligners. It is worth noting that the runtime in the figure is in $\log _{10}$ scaled. The elapsed time scales nonlinearly for a growing number of threads are attributable to the limitation of Amdahl’s law. Particularly, reading and writing large sequenced files gradually becomes the computational bottleneck as threads increase.

Moreover, we assessed the runtime of long-read SV callers (SVIM, cuteSV, and PBSV) when they took the results of Leaf and the aligners as input. We used the default parameters of each SV caller for the assessment. We expected Leaf-SV callers to run faster because Leaf outputs more concise SAM/BAM than aligners for PacBio raw reads. In the results shown in Fig. 7, SVIM is single-threaded and runs approximately $1.75\times$ faster when taking as input the results of Leaf. cuteSV takes as input the results of Leaf runs over $3 \times$ faster. PBSV combined with Leaf runs approximately $1.2\times$ faster using either a single thread or multiple threads. The assessment suggests that filter-based pipelines, such as Leaf-based ones, could be more computationally efficient than conventional pipelines.

## Discussion

We conducted different kinds of assessments in this work to reduce the potential biases caused by the complexity and diversity of long-read SVs. Despite the assessment differences, the results are essentially in accordance with the expectation that Leaf-SV callers can achieve comparable precision while outperforming aligner-SV callers in computational efficiency and sensitivity, particularly for nonlinear intra-read SVs. For instance, the assembly-based assessment for detecting insertions and deletions in HiFi reads is in line with the trio-based assessment. Both the assessments suggest $recall_{Leaf|Aligner}\approx 1$ defined in expression Eq. 1. Namely, most true insertions and deletions detected by aligners can also be detected by Leaf. Additionally, the assembly-based assessment of HiFi read is also in line with the detectable SV space assessment (i.e., sequencing error 0.1 in column INS/DEL Fig. 5). Moreover, both the trio-based assessment and SV space assessment suggest Leaf performs better in detecting nonlinear intra-read SVs such as intra-read inversions. It is in line with the expectation that capturing SV signals at an early stage can enhance the performance of SV detection pipelines.

As a new type of pipeline, limitations exist that could be addressed in the future. Although the outputs of Leaf are compatible with SV callers, the performance of Leaf-SV caller pipelines can be further improved. For instance, endpoints of SV signals reported by Leaf are commonly more divergent than aligners, as shown in Additional File 1: Fig. S2. In consequence, existing alignment-based SV callers are more likely to fail in computing the consensus endpoints of supporting reads. We found in the assessment that a considerable number of SV signals were detected by Leaf but could not be recalled by SV callers due to endpoint divergence. Therefore the performance of Leaf-SV caller pipelines can be further improved by improving the consensus of endpoint. To this end, we can align a short sequence containing endpoints of the SV signals to reduce the endpoint divergence. It is simple to implement and is compatible with existing alignment-based SV callers. Another solution is to develop a brand new filter-based SV caller. Although it would be more complex to implement, the performance of filter-based pipelines would potentially be fully exploited.

## Conclusion

In this work, we proposed a new filter-based pipeline for population-scale long-read SV detection. The core idea of the filter-based pipeline is to capture SV signals at an early stage, which are likely missed by many long-read aligners. To this end, we implemented Leaf and conducted comprehensive assessments in this work, which suggest Leaf has the following features and benefits compared to aligners: First, it is comparable to aligners in terms of mapping insertion and deletion detection. Second, it has an outstanding performance in mapping nonlinear intra-read SVs. Third, it is much more computationally efficient than long-read aligners. Finally, Leaf is a technical validation revealing the feasibility and potential of long-read filter-based pipelines. The performance of the filter-based pipelines can be further improved as a growing number of optimizations are employed.

## Methods

Here, we discuss the main methods employed by Leaf, which consists of four modules:

1. A canonical binning module for quick clustering patterns in long reads. It takes long reads as input and output clustered anchors of matched patterns in the read and the reference.
2. An adversarial autoencoder (AAE) for screening discordant anchors and computing priors of potential SV gaps.
3. A generative module for computing the likelihoods of each potential assembly of anchors and generating the most likely SV mappings.
4. An adaptation module for trimming and adapting the results to the format compatible with SV callers.

### Anchor binning

In the first module, the canonical binning is employed to cluster anchors. We use the refined minimizers [48] as the patterns for binning, which can be briefly described as follows. Denote $$p_{ij}=(h_{ij},x_{ij})$$ as the *j*th pattern sampled from the sequence *i*, where $$h_{ij}$$ is the hash value of the pattern and $$x_{ij}$$ is the position of $$p_{ij}$$. Given two matched patterns $$(p_{gi}$$, $$p_{rj})$$ from genome *g* and read *r*, whose hash values are identical (i.e. $$h_{gi}=h_{rj}$$), denote $$A_{ij}=x_{gi}-x_{rj}$$ as the anchor of $$p_{gi}$$ and $$p_{rj}$$. $$A_{ij}$$ within the given bound are clustered into bins. Specifically, the key to the bin for anchor $$A_{ij}$$ is given by $$key=\lfloor A_{ij}/n\rfloor$$, where constant *n* is the interval of the bin, and $$\lfloor \cdot \rfloor$$ is the floor operator. We built a genome index to speed up the binning process, where anchors are collected by streaming read *r* and querying the index for matched patterns. Bins containing sufficient anchors are then selected for likelihood computation in the next stage.

### AAE for priors

Due to sequencing errors and intra-read SVs of long reads, discordant anchors exist that constitute gaps. We conduct a preliminary screening of discordant anchors at this stage to classify and assign SV priors. The screening results are used to help initiate the generative model in the next stage. Intuitively, the idea of the screening is to generate an overall impression of whether the anchors are likely from SVs and then initiate the generative model by passing a continuous variable, the prior. Without the screening, we may discretely classify a gap $>50\;\mathrm{bps}$ for instance, as a potential indel signal (i.e., prior = 0 or 1), while the screening may assign indel prior, probably 0.6, to a gap of 50 bps. It helps better process intra-read SV signals. The prior function for discordant anchors involves latent relations regarding gap shape and size, etc., which may be hard to define explicitly. A workable solution is to employ a trained network. We implemented an adversarial autoencoder (AAE) prototype to help initialize SV priors for discordant anchors. The AAE implementation is further described in Additional File 1: Section 3.1. It is worth noting that the screening does not generate the final results (i.e., sequence mappings). Instead, it is used to aid the generative model, which generates accurate mappings and is independent of the training data.

### Generative model

We use the generative model to generate the most likely assembly of fragments from which the given read is sequenced. The core idea is to use likelihood [49] functions instead of score functions to compute the optimal assembly of fragments. Intuitively, it is more reasonable to use smooth likelihood functions involving multiple variables for modeling the assembly of fragments.

Denote $a_i$ as the subassembly from which the subread $r_i, i=1,2,..$ is sequenced. Assuming $a_i$ and $r_i$ depend on parameters $\Theta =\{\theta _1,\theta _2..\}$, (e.g., sequencing error *e*, length *l*, and SV *v*) then the likelihood that $r_i$ is sequenced from $a_i$ is given by

$$\begin{aligned} \mathcal {L}(a_i;r_i)=p(r_i|a_i)\approx p(\Theta ) \end{aligned}$$

**Fig. 8:**
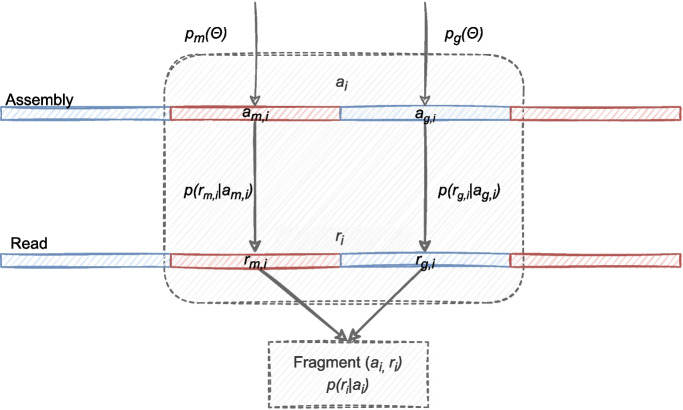
Fragment likelihood $p(r_i|a_i)$ model, which is the probability that subread $r_i$ is sequenced from subassembly $a_i$. The *i*th fragment $(a_i,r_i)$ is dived into independent map $(a_{m,i},r_{m,i})$ (red) and gap $(a_{g,i},r_{g,i})$ (blue). $p(r_i|a_i)$ correspondingly comprises $p(r_{m,i}|a_{m,i})$ and $p(r_{g,i}|a_{g,i})$. The two likelihoods are approximated by $p_{m}$ and $p_{g}$, which are functions of observable variables $\Theta$

Assuming *e*, *l*, and *v* are the main parameters in $\Theta$ and the fragment $(a_i,r_i)$ comprises a subfragment of map $(a_{m,i},r_{m,i})$ and an independent subfragment of SV gap $(a_{g,i},r_{g,i})$ at the $5'$ end, then the likelihood above is approximated by

$$\begin{aligned} p(r_i|a_i){} & {} =p(r_{m,i}|a_{m,i})p(r_{g,i}|a_{g,i})\approx p_m(\Theta )p_g(\Theta ) \\{} & {} \approx p_{m}(e_{m,i},l_{m,i})p_{g}(e_{g,i},l_{g,i},v_{g,i}) \end{aligned}$$

where $p_{m}$ and $p_{g}$ are the map and gap probabilities as shown in Fig. 8. Provided sequencing error *e* is constant for a given read, we use $p_{g,e}(l,v)$ to denote $p_{g}(e,l,v)$ in the following discussion. Assume *v* comprises *n* independent basic SVs (or gap) $v_j\in \{$indel, inversion, duplication, reg$\dots \}$ nested in the gap, where “reg” refers to the regular gap free of any SV. Formally, denote $v=\bigcup _{j=1}^{n}v_j$ the nested SV in *g* then $p_{g,e}(l,v)$ is given by

2 $$\begin{aligned} p_{g,e}(l,v)=p_{g,e}\left(l,\bigcup _{j=1}^{n}v_j\right)=\sum \limits _{j=1}^{n} p_{g,e}\left(l,v_j\right)-\sum \limits _{j=1}^n \sum \limits _{k=1}^{j} p_{g,e}\left(l,v_j\right)p_{g,e}\left(l,v_k\right) +... \end{aligned}$$

We use expression Eq. 2 to integrate an arbitrary number of SVs ($v_j$) into the gap model, while restrictions on coexistence of $v_j$ are further defined in Additional File 1: Table S2. Expression Eq. 1 applies to probabilities of nested SVs as well as single basic ones. For instance, the probability of a basic indel gap can be expressed by simply setting probabilities of reg, inversion and duplication 0 or a small value in expression Eq. 2.

**Fig. 9:**
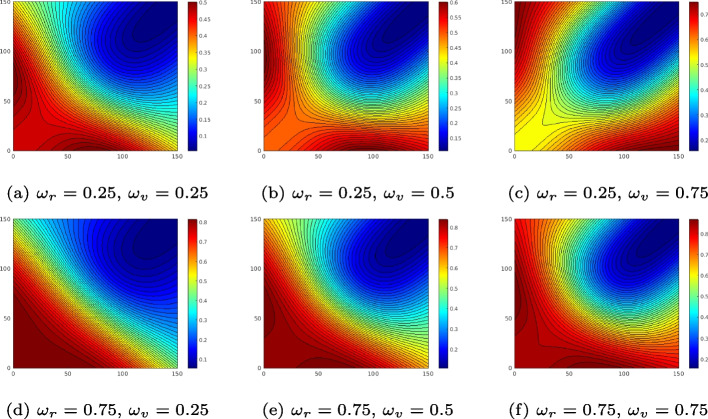
Contour lines of likelihood $p_{g,e}(l_x,l_y,v)\in [0,1]$ defined in expressions Eqs. 2 and 3. Horizontal ($l_x$) and vertical ($l_y$) axes in each subfigure are gap lengths of the assembly and read. $w_r$ and $w_v$ are the priors of regular gaps and SV (indel) gaps

Then we define $p_{g,e}(l,v_j)$ in expression Eq. 2. Assume the gap of *l* in length comprises the gap in the assembly of $l_x$ in length and the gap in the read of $l_y$ in length, then $p_{g,e}(l,v_j)$ is given by

3 $$\begin{aligned} p_{g,e}(l,v_j)=p_{g,e}(v_j)p_{g,e}(l|v_j)=\omega _{v_j} p_{g,e}(l|v_j)=\omega _{v_j}p_{g,e}(l_x,l_y|v_j) \end{aligned}$$

where $\omega _{v_j}=p_{g,e}(v_j)$ is the prior of $v_j$. $p_{g,e}(l_x,l_y|v_j)$ regarding each $v_j$ are defined in Additional File 1: Section 3.2. Plugging $p_{g,e}(l_x,l_y|v_j)$ into expression Eq. 2, we have $p_{g,e}(l_x,l_y,v)$ visualized in Fig. 9, where free variables $\omega _v$ and $\omega _r$ are priors of SVs and regular gap. For instance, for a gap of $l_x=150$ and $l_y=0$, which is likely a deletion of 150*bps* in length, $p_{g,e}$ in subfigure (a) of smaller $\omega _v$ outputs a lower likelihood at point (150, 0), while $p_{g,e}(l_x,l_y, v)$ in subfigure (c) of larger $\omega _v$ outputs a higher likelihood at point (150, 0). Moreover, each subfigure has the lowest probability at point (150, 150), which is likely to be an incorrect gap rather than a regular gap or SV gap. It is worth noting that models for $p_{g,e}(l|v_j)$ are not limited to the ones in the Additional File 1: Section 3.2. For instance, variables $\theta _i\in \Theta$ other than *l* possibly better fit the SV probabilities (i.e. $p_{g,e}(\Theta |v_j)$).

Finally, we compute the most likely assembly denoted by $\hat{A}_i$ for $r_1,..,r_i$. Assuming each $r_i$ is sequenced independently from the assembly, the likelihood of at least one subsequence $r_1,r_2,...,r_i$ being sequenced from $A_i$ is given by

4 $$\begin{aligned} \mathcal {L}_i=\mathcal {L}(A_i;r_1,r_2,...,r_i) =\left( 1-p(r_i|a_i)\right) \cdot \mathcal {L}_{i-1} + p(r_i|a_i) \end{aligned}$$

$\mathcal {L}_i$ is computed by dynamic programming (DP) and $\hat{A}_i$ is the one that maximizes $\mathcal {L}_i$.

### Adaptation

#### Extended SAM/BAM

The standard SAM/BAM is a widely used alignment format. We adapt the results of the generative model to SAM/BAM by extending the meaning of the columns in the standard SAM/BAM format. The standard SAM/BAM records base-to-base alignment, which is a pair of two identical bases whose positions in the sequences can be denoted by a tuple of (*x*, *y*). However, the concept of identical bases does not apply to the results of the generative model, whose bases involve the concept of likelihood. To this end, we extended the meaning of SAM/BAM by introducing the concept of deviation *d* to the tuple, namely $(x,y)\rightarrow (x,\hat{y},d)$, where $d=y-\hat{y}$ is the deviation between base *y* and its estimation denoted by $\hat{y}$. Since *d* is commonly randomly varied, we denote *D* as the random variable over all possible *d* and use tuple $(x,\hat{y}, D)$ to express the results of the generative model. To record tuple $(x,\hat{y}, D)$, we employ the cigar string of the standard SAM/BAM to record $(x,\hat{y})$, while it is not necessary to explicitly record *D* for each cigar since it commonly follows a distribution determined by the model. For instance, in the case of the generative model, if *D* follows a normal distribution suggesting *x* is likely to be sequenced from one of the bases around $\hat{y}$ then we can record the mean $\mu$ and variance $\sigma ^2$ in the headers or operational fields of SAM/BAM. Therefore, existing fields in the standard SAM/BAM are sufficient for recording the tuple $(x,\hat{y}, D)$. Exact definitions for each field in the extended SAM/BAM are discussed in Additional File 1: Section 3.3. Finally, it is worth noting that standard SAM/BAM of alignment is a special case of extended SAM/BAM discussed above, where *x* is regarded as always sequenced from $\hat{y}$ (i.e., $D\equiv 0$). Therefore, the definition above is an extension of the standard SAM/BAM and it applies to alignments as well.

#### Software compatibility 

We tested the compatibility of the extended SAM/BAM for the following toolkits. In principle, they can be directly combined with Leaf without adjustment. However, the interpretation of the toolkit output should be changed correspondingly when taking extended SAM/BAM as input. Especially, the deviation concept should always be taken into account when applying the extended SAM/BAM to them.

1. SAMtools version 1.10: It is the toolkit for SAM/BAM operations. Three SAMtools modules used in this work were tested. They are SAMtools view, SAMtools index, and SAMtools sort for viewing and indexing SAM/BAM.
2. IGV version 2.8.3: It is the sequence visualization tool. The results of Leaf can be visualized directly by IGV. It is also worth noting that gaps shorter than the deviation, commonly $<50\;\mathrm{bps}$, in the visualized results should be ignored since they are insignificant.
3. PBSV version 2.3.0: PBSV is an SV caller for PacBio sequencing reads. It takes SAM/BAM files as input. The sample name and read group should be specified in the SAM header when using PBSV. The compatibility of Leaf with PBSV was tested. The results can be processed directly by PBSV with default settings.
4. SVIM version 1.2.0: SVIM is an SV caller for PacBio and ONT reads. It takes the SAM/BAM as input. The compatibility of Leaf with SVIM was tested. Leaf results can be processed directly by SVIM. It is better to use the default arguments for either PacBio CLR or HiFi reads when combined with Leaf due to the deviations discussed above.
5. cuteSV version 1.0.13: cuteSV is an SV caller for PacBio and ONT reads. It takes as input the SAM/BAM as well. The compatibility of Leaf with cuteSV was tested. Leaf results can be processed directly by cuteSV. We also suggest to use default arguments for different types of long reads.

### Supplementary information

Additional file 1. Supplementary figures, tables and methods.

Additional file 2. Peer review history.

## Data Availability

Leaf source code is available at https://github.com/xp3i4/linear under the BSD license. An archived version is available on Zenodo [50]. Datasets used in the trio-based assessment include: Ashkenazim Jewish trio [37, 38], Han Chinese trio [37], whose sequencing data is available at GIAB ftp site ftp://ftp-trace.ncbi.nlm.nih.gov/giab or NCBI SRA linked to PRJNA200694. SKBR3 breast cancer cell line dataset is available in study [39], whose sequencing data is available on NCBI BioProject linked to PRJNA476239. Datasets for SV space assessment are available on Zenodo [51]. Datasets used in the assembly-based assessment include HPRC diploid assembly of HG00733, which is available in study [52] (https://github.com/human-pangenomics). HG00733 PacBio HiFi reads is a dataset in study [53], whose sequencing data is available at site https://s3-us-west-2.amazonaws.com/human-pangenomics/index.html or NCBI SRA with accession number ERX3831682.
