# Supplementary material for: Leaf: an ultrafast filter for population-scale long-read SV detection
Source: Genome Biol. 2024 Jun 13;25:155. doi: 10.1186/s13059-024-03297-5 (PMC11170821; doi:10.1186/s13059-024-03297-5)
Supplement: Supplementary file 1 — Additional file 1. Supplementary figures, tables and methods. [file 13059_2024_3297_MOESM1_ESM.pdf]

Leaf: an ultrafast filter for population-scale long-read SV detection

Chenxu Pan[1] and Knut Reinert[1,2]

[1]*Department of Mathematics and Computer Science, Free University of Berlin, Takustr. 9, 14195 Berlin, Germany*
[2]*Department of Computational Molecular Biology, Max Planck Institute for Molecular Genetics, Berlin 14195, Germany*

Contents

1 Supplementary Figures **2**

2 Supplementary Tables **3**

3 Supplementary Methods **5**

3.1 AAE for SV priors . . . 5

3.1.1 Model . . . 5

3.1.2 Data . . . 6

3.2 Generative models for SVs . . . 8

3.3 Extended SAM/BAM definition . . . 9

3.3.1 Sections . . . 9

3.3.2 Cigar string of SAM* . . . 9

3.3.3 SEQ of SAM* . . . 9

3.4 Software version . . . 11

1 Supplementary Figures

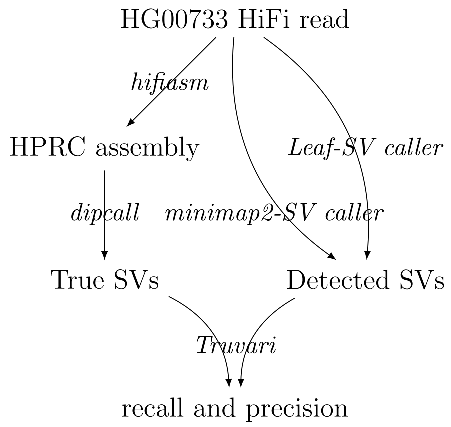

Fig S1: Datasets and tools used in the assembly-based assessment. Tools are marked with italic text. hifiasm and dipcall are minimap2-based. Truvari is a benchmark tool to compare SVs.

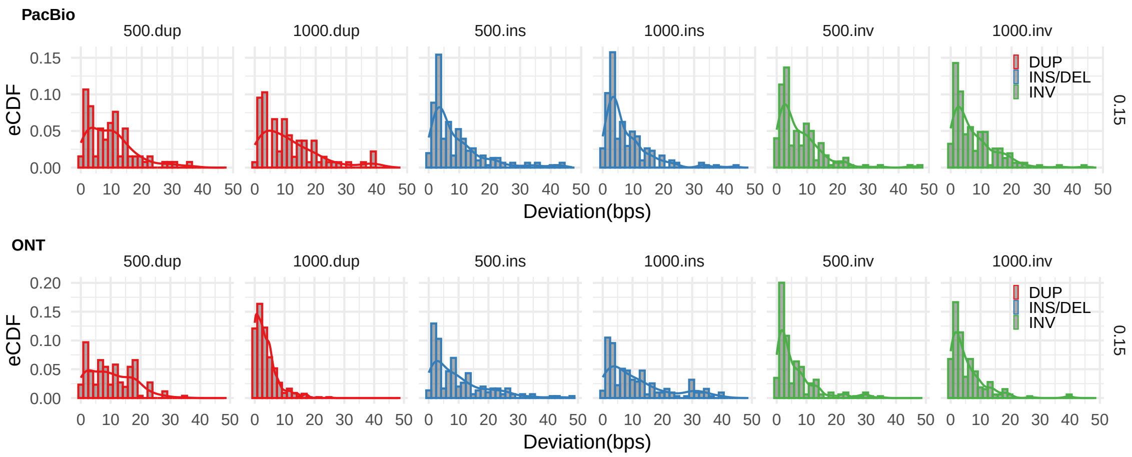

Fig S2: Detectable SV space assessment: Empirical distribution of deviations of SV endpoint detected by Leaf for SV length=500,1000bps and sequencing error=0.15

2 Supplementary Tables

Table S1: $recall_{Leaf-SVcaller|minimap2-SVcaller}$ and $recall_{NGMLR-SVcaller|NGMLR-SVcaller}$ for supporting reads (2-16) in the assembly-based assessment.

		2	4	6	8	10	12	14	16
cuteSV	Leaf	0.9909	0.9684	0.9552	0.9433	0.9311	0.9173	0.9052	0.8881
	NGMLR	0.7596	0.7587	0.7545	0.7446	0.7438	0.7345	0.7160	0.6943
SVIM	Leaf	0.9986	0.9488	0.9135	0.8895	0.8685	0.8467	0.8376	0.8193
	NGMLR	0.7278	0.7266	0.7088	0.6972	0.6885	0.6857	0.6833	0.6741

Table S2: Truth table if SV gaps (v_j) can be nested in a fragment gap in the generative model.

v_j	INS	DEL	INV	DUP	Regular
INS	-	False	True	False	True
DEL	-	-	True	True	True
INV	-	-	-	True	True
DUP	-	-	-	-	True
Regular	-	-	-	-	-

Table S3: Header section supported by SAM*. Header lines with ✓ are identical to SAM_0^*.

Header	Description	Support
@HD	File-level metadata. Optional.If present, there must be only one @HD line and it must be the first line of the file.	✓
@SQ	Reference sequence dictionary. The order of @SQ lines defines the alignment sorting order.	✓
@RG	Read group. Unordered multiple @RG lines are allowed.	✓
@PG	Program.	✓
@CO	One-line text comment. Unordered multiple @CO lines are allowed. UTF-8 encoding may be used.	✓

Table S4: Cols in alignment section supported by SAM*. Cols with ✓are identical to SAM_0^*.

Col	Field	Description	Support
1	QNAME	Query template NAME	✓
2	FLAG	Bitwise FLAG	✓
3	RNAME	Reference sequence NAME	✓
4	POS	1-based leftmost POSition	✓
5	MAPQ	MAPping Quality	✓
6	CIGAR	CIGAR string	Extended
7	RNEXT	Reference name of the mate/next read	✓
8	PNEXT	Position of the mate/next read	✓
9	TLEN	Observed Template LENgth	✓
10	SEQ	Segment SEQuence	Extended
11	QUAL	ASCII of Phred-scaled base QUALity	✓
12	TAG	Optional tags	Extended

Table S5: Cigar operations supported by extended SAM*. Operations with ✓are identical to SAM_0^*.

Cigar	Operation	Description	Support
M	Match	Match. This could contain two different letters (mismatch) or two identical letters.	✓
D	Deletion k	Deletion (gap in the target sequence).	✓
I	Insertion	Insertion (gap in the query sequence).	✓
N	None	skipped region from the reference	✓
S	Soft clipping	Segment of the query sequence that does not appear in the alignment. This is used with soft clipping, where the full-length query sequence is given (field 10 in the SAM record). In this case, S operations specify segments at the start and/or end of the query that do not appear in a local alignment.	✓
H	Hard clipping	Segment of the query sequence that does not appear in the alignment. This is used with hard clipping, where only the aligned segment of the query sequences is given (field 10 in the SAM record). In this case, H operations specify segments at the start and/or end of the query that do not appear in the SAM record.	✓
=	Match	Alignment column containing two identical letters.	Extended
X	Mismatch	Alignment column containing a mismatch, i.e. two different letters.	Extended

Table S6: SEQ* alphabets corresponding to cigars, where 1/0 means the SEQ* alphabet is/is not equal to the corresponding ith or jth alphabet of read or reference denoted by rd_i and rf_j. 'N' means the alphabet SEQ* is 'N'.

	=	X	M	I	D	S	H
read(rd_i)	0	if $rd_i \neq rf_j$ then rd_i	0	1	0	1	0
ref(rf_j)	1	if $rd_i = rf_j$ then N	1	0	0	0	0

3 Supplementary Methods

3.1 AAE for SV priors

3.1.1 Model

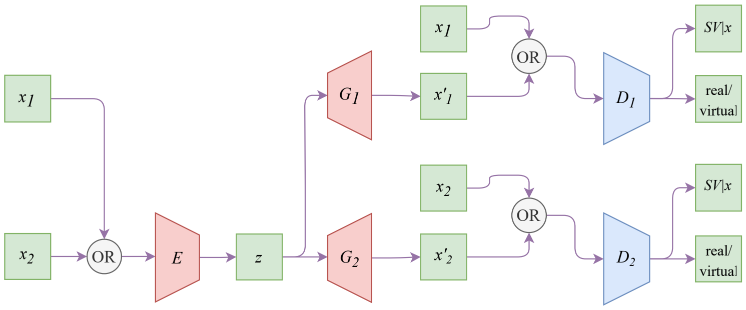

Fig S3: The structure of the AAE for SV prior computation. E is the encoder extracting features of inputs, which are anchors of bins (x_1) and anchors of alignment (x_2), to the common latent space z. G_i is the generator, which reconstructs z as virtual anchors x_i'. x_i' with real anchors x_i are used to train the discriminator D_i. D_i emits the probability of if the input anchors are real and the SV prior (i.e. $p(SV|x)$). G_i, D_i compete against each other for training to generates x_i' resembling x_i.

We defined the AAE to compute SV priors for a given set of anchors. AAE helps better process latent signals in patterns.

$$Anchors \xrightarrow{AAE} SV\ priors$$

Figure S3 shows the AAE structure. We input anchors in the selected bins (x_1) and anchors sampled from the corresponding alignment of the read (x_2) for training. The encoder E extracts the main features of x_1 and x_2 to the common latent space denoted by $z = E(x)$. The generator G_1 generates virtual anchors of bins denoted by $x_1' = G_1(z)$. The generator G_2 generates virtual anchors of alignment denoted by $x_2' = G_2(z)$. Then we use the virtual anchors of bins x_1' combined with the real anchors of bins x_1 to fool the discriminator D_1. Similarly, we use x_2' combined with x_2 to fool the discriminator D_2. D_1 and D_2 learn to discriminate the real and virtual anchors and output SV priors. D_1, D_2 and G_1, G_2 compete with each other to generate virtual anchors x_1', x_2' that resembles the real ones x_1, x_2.

There exist two optional pipelines to compute SV priors for the given anchors of bins x_1. In the first one, we input anchors of bins x_1 to D_1 and D_1 outputs SV priors.

$$x_1 \rightarrow D_1 \rightarrow priors$$

In the second one, we input anchors of bins x_1 to the encoder E. E extracts features to z. G_2 then generates virtual anchors of alignment from z and pass it to D_2. D_2 then outputs the priors.

$$x_1 \rightarrow E \rightarrow G_2 \rightarrow D_2 \rightarrow priors$$

Clearly, the first one is faster to compute. The strategy of choosing the pipelines is as follows. We run the first pipeline if the SV types for the anchors cannot be directly determined. If returned priors are insignificant then we again run the second one.

$$Directly\ identify \xrightarrow{Succeed} priors$$
$$| \xrightarrow{Fail} AAE\ pipeline\ one \xrightarrow{Succeed} priors$$
$$| \xrightarrow{Fail} AAE\ pipeline\ two \rightarrow priors$$

It is worth noting that the priors returned by the AAE are a kind of subjective assessment rather than the ground truth.

The loss functions of the AAE described above are defined as follows. The training is to minimize the generic loss which is given by

$$\min \mathcal{L}_{AAE} = \lambda_1 \mathcal{L}_E + \lambda_2 \sum_{i=1}^{n} \mathcal{L}_{G_i} - \lambda_3 \sum_{i=1}^{n} \mathcal{L}_{adv_i} + \lambda_4 \sum_{i=1}^{n} \mathcal{L}_{D_i}$$

where λ_i are learning rates, $\mathcal{L}_E$, $\mathcal{L}_{G_i}$, $\mathcal{L}_{adv_i}$, $\mathcal{L}_{D_i}$ are the loss of the encoder, the loss of the ith generator, the adversarial loss of the ith generator and the loss of the ith discriminator.

Given the training data x_i, $i \in \{1, 2\}$ in the AAE, and the corresponding type of SVs denoted by SV_i, the loss of the ith generator is given by the distance between the real and virtual data

$$\mathcal{L}_{G_i} = \mathbb{E}_{j=1,2,..}[||G_i \cdot E \cdot x_j - x_j||^2]$$

where $\mathbb{E}_{p(x)}[\cdot]$ is the expected value operator $\int p(x) \cdot dx$ with respect to the distribution $x \sim p(x)$. $G_i \cdot E \cdot x_j$ is the virtual data generated by G_i. Namely input x_j to encoder E, which outputs z, and generator G_i takes z as input. The virtual data generated by a trained G_i is supposed to resemble the real one x_j. Thus the distance between the virtual data and real data are used as the loss.

The adversarial loss of the ith generator is given by

$$\begin{aligned}\mathcal{L}_{adv_i} =& \sum_{j=1}^{n} \mathbb{E}[\log p\,(SV_i|G_i \cdot E \cdot x_j)] - \sum_{j=1}^{n} \mathbb{E}[\log p\,(virtual|G_i \cdot E \cdot x_j)] + \\ & \sum_{j \neq i} \mathbb{E}[\log p\,(SV_i|G_i \cdot E \cdot G_j \cdot E \cdot x_i)] - \sum_{j \neq i} \mathbb{E}[\log p\,(virtual|G_i \cdot E \cdot G_j \cdot E \cdot x_i)]\end{aligned}$$

All virtual data identified by the discriminator are backprogate as the penalty, the $-\mathbb{E}[\cdot]$ on the right side of the expression. G_i and D_i only compete for virtual or real data, while they don't compete against each other for SVs types. Namely G_i don't fool D_i for SVs type. Thus the left side of the expression is to enhance the capability of classifying the type of SV.

The loss of the ith discriminator D_i is given by

$$\begin{aligned}\mathcal{L}_{D_i} =& \mathbb{E}[\log p(real|x_i)] + \mathbb{E}[\log p(SV_i|x_i)] + \\ & \sum_{j \neq i} \mathbb{E}[\log p\,(virtual|G_i \cdot E \cdot x_j)] + \sum_{j \neq i} \mathbb{E}[\log p\,(SV_i|G_i \cdot E \cdot x_j)]\end{aligned}$$

The first line of $\mathcal{L}_{D_i}$ is to identify the real data x_i. The second line is to enhance the capability of discriminating virtual data from x_j.

3.1.2 Data

Reads and SVs The AAE in this work is trained using PacBio CLR reads in HG002 Ashkenazim Jewish trio from GIAB project. Alignments of centromeres are excluded. We used RSVSim to simulate SVs and planted them randomly in each read to generate intra-read SVs. SVs are randomly planted and thus are allowed to be nested in one read. Each read is planted by two simulated SVs at most. Inter-read SVs, which are SVs between reads, are supposed to be resolved by SV callers. Therefore, they are not simulated in the training datasets.

Alignments Reads planted with SVs are split into two groups, subsequences free of SVs and subsequences of SVs. They are aligned separately, since aligners may falsely align the SV subsequences. Alignments are classified into three types.

1. Linear alignment: Alignment that does not contain SVs.
2. Alignment of SVs: Alignment containing intra-read SVs.
3. Forced alignment: Alignment of two random sequences.

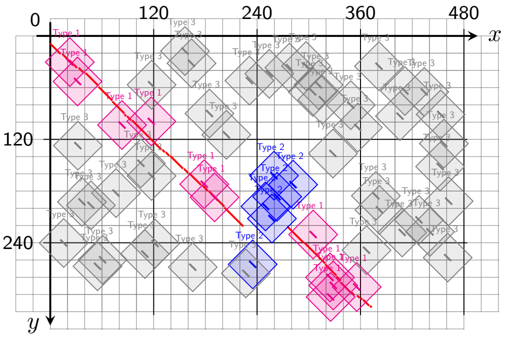

Fig S4: An example of labeled anchors spanning a deletion. Binning anchors x_1 of type 1,2,3 with fixed edit distance regions around the anchors are labeled with rectangles. Red lines are alignment anchors x_2. Axes are the given sequences.

Anchors Anchors of alignment (x_2) are collected based on SAM files of the three types of alignments discussed above. Anchors of binning (x_1) are collected and classified into the following three types correspondingly, as shown in figure S4.

1. Anchor around linear alignment: If there exists linear alignment around anchors within the given edit distance bound.

2. Anchor around alignment of SVs: If it is not type 1 anchor and there exists alignment of SVs around anchors within the given edit distance bound.

3. Anchor of forced alignment, otherwise.

For simplicity, the output of D_1 for training is a 2-dimensional 0-1 vector indicating if anchors of bins are around an SV and if the anchors are real or virtual. The output of D_2 for training is a 2 dimensional 0-1 vector indicating if anchors of alignment are from SVs and if the anchors are real or virtual.

3.2 Generative models for SVs

We define the empirical distributions of each SV type $p_{g,e}(l_x, l_y|v_j)$ as follows.

Regular gap Denote v_r the regular gap which does not have SVs. Since $l \geqslant 0$, we use the CDF of gamma distribution in expression 1 to model $p(l_x, l_y|v_r)$.

$$\begin{cases} F(x; \alpha, \beta) = \dfrac{\gamma(\alpha, \beta x)}{\gamma(\alpha, \infty)} \\ \gamma(a, x) = \displaystyle\int_0^x t^{a-1} e^{-t} dt \end{cases} \tag{1}$$

Assuming pior $p(v_r) = \omega_r$, then $p(l, v_r)$ is given by

$$p(l_x, l_y, v_r) = p(l_x, l_y|v_r)p(v_r) = \omega_r \left(1 - F\left(\min(|l_x|, |l_y|), \alpha_r, \beta_r\right)\right)$$

Insertion and deletion Since the insertion and the deletion cannot coexist in one gap, we compute the likelihood of indel $p_{l,indel}$, which is given by

$$p_{l,indel} = p_{l,ins}(1 - p_{l,del}) + p_{l,del}(1 - p_{l,ins}) \tag{2}$$

where $p_{l,ins}$ or $p_{l,del}$ is the probability of a gap is an insertion or a deletion of length l. Denote l_x and l_y the gap in the assembly and read, and $l_x - l_y \in (-\infty, +\infty)$. We use the normal distribution for $p_{l_x,l_y|ins}$ and $p_{l_x,l_y|del}$. Assuming priors $p_{ins} = \omega_{v,ins}$ and $p_{del} = \omega_{v,del}$, then $p_{l_x,l_y,ins}$, $p_{l_x,l_y,del}$ are given by

$$\begin{cases} p_{l_x,l_y,ins} = p_{ins} p_{l_x,l_y|ins} = \omega_{v,ins} \Phi(l_x - l_y; \mu_1, \sigma_1) \\ p_{l_x,l_y,del} = p_{del} p_{l_x,l_y|del} = \omega_{v,del} \Phi(l_x - l_y; \mu_2, \sigma_2) \end{cases} \tag{3}$$

where Φ is the CDF of normal distribution, μ_1 μ_2 and σ_1 σ_1 are means and standard deviations of $l_x - l_y$ for insertion and deletion.

Tandem duplication We regard (tandem) duplication as one specific type of insertions, whose $l_x - l_y \in (-\infty, \infty)$, $l_x \leqslant 0$ and $l_y \geqslant 0$. Hence we also apply the normal distribution to model $p_{l_x,l_y,dup}$. Assuming the prior $p_{dup} = \omega_{v,dup}$, $p_{l_x,l_y,dup}$ is given by

$$p_{l_x,l_y,dup} = p_{dup} p_{l_x,l_y|dup} = \omega_{v,dup} \Phi(l_x, -l_y, \mu_3, \sigma_3)$$

Inversion Since $l_x,\ l_y \geqslant 0$, we use the gamma distribution to model $p_{l_x,l_y,inv}$. Assuming $p_{inv} = \omega_{v,inv}$, $p_{l_x,l_y,inv}$ is given by

$$\begin{aligned} p_{l_x,l_y,inv} &= p_{lx,ly|inv} p_{inv} \\ &= \omega_{v,inv} \left(1 - F(l_x + l_y; \alpha_{inv}, \beta_{inv})\right) \end{aligned} \tag{4}$$

Map Assuming the length of the read is L, we use variable l/L to compute the likelihood of map $p_m(l)$. The Beta family is theoretically better for $l/L \in [0, 1]$ though. For simplicity, we compute $p_{l_x,l_y,map}$ by

$$p_{l_x,l_y,map} = p_{l_x,l_y|reg} p_{reg} = \omega_{map} \frac{\min(l_x, l_y)}{L}$$

where ω_{map} is the prior of map.

The likelihood of an SV gap is computed by the following expression, where v_j is one of the basic SVs, including the insertion, deletion, duplication and inversion discussed above.

$$p_{g,e}(l, v) = p_{g,e}(l, \bigcup_{j=1}^{n} v_j) = \sum_{j=1}^{n} p_{g,e}(l, v_j) - \sum_{j=1}^{n} \sum_{k=1}^{j} p_{g,e}(l, v_j) p_{g,e}(l, v_k) + ...$$

Table S2 shows how different v_j are allowed to be nested in the gap model.

3.3 Extended SAM/BAM definition

3.3.1 Sections

The Sequence Alignment/Map (SAM) format `https://github.com/samtools/hts-specs` is a TAB-delimited text format comprising an optional header section and an alignment section. We denote SAM^* as the extended SAM and SAM_0^* as the standard SAM in the following text. In the extension, SAM_0^* is a subset of SAM^*, which supports the alignments and Leaf results. Namely, SAM^* and SAM_0^* of alignments are identical. The SAM/BAM standard `https://samtools.github.io/hts-specs/SAMv1.pdf` (SAM_0^*) comprises two sections:

* **The header section:**
  Each line is TAB-delimited and, apart from @CO lines, each data field follows a format 'TAG:VALUE' where TAG is a two-character string that defines the format and content of VALUE.

* **The alignment section:**
  The alignment section of SAM_0^* typically represents the linear alignment of a segment. Each line comprises 11 mandatory or more TAB-separated fields.

SAM^* inherits the header and alignment section of SAM_0^* and extends several fields in the two sections. Table S3 and table S4 show the header section and the alignment section supported in SAM^*.

3.3.2 Cigar string of SAM^*

We use the cigar string to record a sequence of $(x_i, \hat{y_i})$. Different from SAM_0^* for recording every base in the alignment (i.e. $x_i - x_{i-1} = 1$), SAM^* does not record every base (i.e. $x_i - x_{i-1} \geqslant 1$). Thus we need to define the cigar string for bases between x_{i-1} and x_i. The cigar string in SAM^* is a string of cigar pairs, which defines a virtual alignment between x_{i-1} and x_i. Formally, they are defined as follows:

Given points $A = (x_1, y_1)$, $B = (x_2, y_2)$ of $x_1 \leqslant x_2$ and $y_1 \leqslant y_2$, the virtual alignment from A to B denoted by $\overline{AB}$ is defined as

$$\overline{AB} = c_M M |c_G| G$$

where cigar counts c_M, $|c_G|$ are given by

$$\begin{cases} c_M = \min(x_2 - x_1, y_2 - y_1) \\ c_G = x_2 - x_1 - y_2 + y_1 \end{cases}$$

And cigar operations M and G are given by

$$M \in \{=, X, M\};\ G = \begin{cases} I; & if\ c_G \leqslant 0 \\ D; & if\ c_G > 0 \end{cases}$$

Intuitively, the expression above defines four types of basic virtual alignment as shown in figure S5. It is worth noting that SAM^* cigar supports alignment since SAM_0^* cigar is a special SAM^* cigar, where each cigar pair satisfies $c_M + |c_G| = 1$.

3.3.3 SEQ of SAM^*

The 10th column of SAM_0^* SEQ records the subsequence of the read. It leads to discordant bases between reads if we apply the SEQ definition to SAM^*. Therefore the SEQ in SAM_0^* is extended. Denote SEQ^*, SEQ_0^* the SEQ of SAM^* and SAM_0^*. SEQ^* is defined as the virtual subsequence of the read that is generated based on the cigar strings, read and reference. Our definition for generating SEQ^* is shown in table S6. Specifically, for the cigar operation $'='$ and $'M'$, the corresponding bases in the reference are inserted into the SEQ^*. For the cigar operation $'X'$, corresponding bases

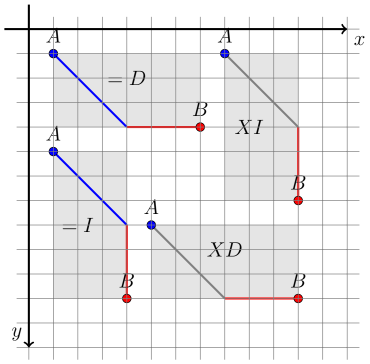

Fig S5: Cigar pairs, $=I$, $=D$, XI, and XD in SAM*. Axes are the reference and read.

in the read are inserted if the base in the read is not equal to the corresponding base in the reference otherwise, base $'N'$ is inserted. For cigar operations of $'I'$ and $'S'$, the corresponding bases in the read are inserted. The definition is to make the inferred reference genomes by different records in the SAM/BAM identical. It is also worth noting that 1. SEQ* is a virtual sequence that is not equal to the subsequence of the read or reference, and thus it cannot be used for recovering the read bases. However, 2. if cigar operation 'M' is not used in the cigar string, then SEQ*=SEQ_0^* holds for alignment. Thus SEQ* supports alignment as well.

3.4 Software version

Table S7: Software used in 1.trio-based 2.detectable SV space 3.assembly-based 4.computational efficiency assessments.

Software	Description	Version	Assessment
minimap2	Enhanced aligner for short and long reads	v2.24	1-4
NGMLR	Long read aligner optimized for variants	v2.7	2-4
PBSIM	PacBio reads simulator	v1.0	2
NanoSim	Nanopore reads simulator	v1.1.0.3	2
PBSV	SV caller for PacBio reads	v2.6.2	4
cuteSV	SV caller for long reads	v1.0.13	1,3,4
SVIM	SV caller for long reads	v1.2.0	1,3,4
IGV	Sequences visualization	v2.8.3	1
SeqAn	Sequences analysis library	v2.0.1	1-4
RSVSim	R package for simulation of SVs	v1.34	2
samtools	Tools for operating SAM/BAM	v1.10	1,3,4
dipcall	A pipeline for calling SVs in assembly-reference alignment	v1.0	3
Truvari	Toolkit for benchmarking, merging, and annotating SVs	v3.0.0	3
