# Supplementary material for: Leaf: an ultrafast filter for population-scale long-read SV detection
Source: Genome Biol. 2024 Jun 13;25:155. doi: 10.1186/s13059-024-03297-5 (PMC11170821; doi:10.1186/s13059-024-03297-5)
Supplement: Supplementary file 2 — Additional file 2. Peer review history. [file 13059_2024_3297_MOESM2_ESM.docx]

**Review history**

**First round of review**

**Reviewer 1**

The authors present a new filter-based pipeline for long read SV calling that generates a set of most likely SV candidates along with the most likely assembly of SVs. The pipeline was demonstrated to improve sensitivity while minimizing the loss of precision when compared with other assembly and alignment based methods. Furthermore, the pipeline offers computational performance improvements over alignment based methods.

Major Notes
I was unable to compile the tool as described on my Mac. Here is the compile error: Could NOT find OpenMP_C (missing: OpenMP_C_FLAGS OpenMP_C_LIB_NAMES)

The grammar is difficult to parse at various points throughout the manuscript. I don't often suggest this because I don't want to come off as rude, but I think that the authors need to go over the manuscript with an editor to significantly improve the English grammar and the flow of ideas.

Nowhere in the manuscript was there any discussion of what was used to train the models. We need to know:
What samples -- real or simulated were used?
What regions of the genome were used for train/test purposes?
Did you explicitly hold out regions of the genome for training/testing?
This goes back to my earlier comment on the AAE, but is also related to the grammar/flow issues of the manuscript:
We need a better description of what the nature of the input/output is at each stage of the pipeline. Be as explicit as possible.
It's all well and good to describe your stages in with mathematical notation/rigour, but in the end other scientists/bioinformaticians have to be able to read it and understand what's actually going on under the hood.

Minor Notes
Nowhere in the manuscript was the name of the pipeline mentioned. The authors refer to it simply as "the filter-based pipeline". It is, however, referred to in one of the figure legends (figure 6).
Figure 2: venn diagrams with more than 3 set intersections are pretty difficult to parse. It would be better to just report some notable intersections and move the figure to the supplementary information.
The description of the adversarial autoencoder is too focused on the internals of the model. For details of the model itself, I would seek out other literature anyway, so we only really need a brief discussion regarding the input/output relationships of the model in the context of the pipeline

Conclusions
The methods described in this article seems like a useful tool for improving/speeding up SV calling pipelines, but there are a few major issues described above that need to be addressed. I wont trust a tool until I have a good understanding of what data was used to train it and at least a rudimentary understanding of the input/output relationships of each stage of the pipeline. I think the notation used in the methods really get in the way of understanding what's going on.

**Reviewer 2**

The authors present a method to perform preprocessing of reads (filter) improve SV calling for long reads. In certain samples and conditions it is stated to improve metrics such as the F1 score.

Unfortunately, there are many critical issues with this study that prevent publication, at least in current form ranging from how the methods were evaluated to how the method is described:

Evaluation:
The methods used to evaluate are not adequate. In particular, there are truth sets that have had considerable effort, and methods developed to compare variant callsets. Genome in a bottle + Truvari is the gold standard, however other methods such as TT-Mars offer a complementary approach.

Compare to Genome In A Bottle truth set (HG002). The number of variants per sample has a huge range, for example between 17774-28924 for HG002, for variants that are >= 80 bps. Even the low number is likely an overestimate as most datasets have ~22k calls for this individual starting at 50 bases, and the majority of SV are short.

At the least, describe how variants are compared (overlap? reciprocal overlap? etc.).

What does it mean for a read to be supporting an SV?

I have many comments about the structure of the manuscript, and writing in general. However, the review took a long time because I tried running a benchmark of linear and did not get positive results relative to simply running minimap2. One potential reason for this is that I used a gold standard variant callset (dipcall on a haplotype-resolved assembly), and only evaluated calls in the euchromatic regions of the genome. The trio analysis could suffer from problems of propagating errors in the parents and child.


I tried the following approach to evaluate linear: variants were called from the HPRC diploid assembly of HG00733
https://github.com/human-pangenomics/HPP_Year1_Assemblies/blob/main/assembly_index/Year1_assemblies_v2_genbank.index


The raw data for the assembly is available here:
https://s3-us-west-2.amazonaws.com/human-pangenomics/index.html?prefix=submissions/8ced218e-9699-458e-9708-eef6969a8065--EXTRAMURAL_SAMPLES/HG00733/hifi/

I called variants from hg38 using dipcall on the assembly. Assembly based SV calls have been shown to be extremely accurate, and a drop in for genome in a bottle truth set.
The change from default was to use a different -z option:
MM2_OPT=-xasm5 --cs -t$(N_THREADS) -z200000,10000

Only calls in the following regions were used:
chr1 5000000 121700000
chr1 125100000 243956422
chr10 5000000 38000000
chr10 41600000 128797422
chr11 5000000 51000000
chr11 55800000 130086622
chr12 5000000 33200000
chr12 37800000 128275309
chr13 5000000 16500000
chr13 18900000 109364328
chr14 5000000 16100000
chr14 18200000 102043718
chr15 5000000 17500000
chr15 20500000 96991189
chr16 5000000 35300000
chr16 38400000 85338345
chr17 5000000 22700000
chr17 27400000 78257441
chr18 5000000 15400000
chr18 21500000 75373285
chr19 5000000 24200000
chr19 28100000 53617616
chr2 5000000 91800000
chr2 96000000 237193529
chr20 5000000 25700000
chr20 30400000 59444167
chr21 5000000 10900000
chr21 13000000 41709983
chr22 5000000 13700000
chr22 17400000 45818468
chr3 5000000 87800000
chr3 94000000 193295559
chr4 5000000 48200000
chr4 51800000 185214555
chr5 5000000 46100000
chr5 51400000 176538259
chr6 5000000 58500000
chr6 62600000 165805979
chr7 5000000 58100000
chr7 62100000 154345973
chr8 5000000 43200000
chr8 47200000 140138636
chr9 5000000 42200000
chr9 45500000 133394717
chrEBV 0 171823
chrM 0 16569
chrX 5000000 58100000
chrX 63800000 151040895
chrY 5000000 10300000
chrY 10600000 52227415


truvari anno svinfo HG00733_dipcall.dip.vcf.gz | bgzip -c > HG00733_dipcall.dip.tv-anno.vcf.gz

bcftools norm -m - HG00733_dipcall.dip.tv-anno.vcf.gz > HG00733_dipcall.dip.tv-anno.norm.vcf.gz
bedtools intersect -header -a HG00733_dipcall.dip.tv-anno.norm.vcf.gz -b hg38.euchromatin.bed -u | bgzip -c > HG00733_dipcall.dip.tv-anno.norm.euch.vcf.gz

Next, reads were aligned using minimap2 and preprocessed with linear.
zcat $DIRC/projects/HPRC/Assemblies_v1/HG00733/*.gz | minimap2 $hg38na/hg38.no_alts.fasta - -x map-hifi -a -t 30 | samtools sort -m6G -@2 -o HG00733.bam
samtools index -@2 HG00733.bam

#!/usr/bin/env bash
linear $DIRC/projects/HPRC/Assemblies_v1/HG00733/*.gz x $hg38na/hg38.no_alts.fasta -ot 3 -o HG00733.linear -rg hgsvg -sn "HG00733" -t 24


Variants were called using cutesv:

#!/usr/bin/env bash
cuteSV --genotype --max_cluster_bias_INS 1000 --diff_ratio_merging_INS 0.9 --diff_ratio_merging_DEL 0.5 -t 8 HG00733.linear.bam hg38.no_alts.fasta HG00733.linear.cutesv.vcf HG00733.linear.cutesv
#!/usr/bin/env bash
conda activate
cuteSV --genotype --max_cluster_bias_INS 1000 --diff_ratio_merging_INS 0.9 --diff_ratio_merging_DEL 0.5 -t 8 ./HG00733.bam hg38.no_alts.fasta HG00733.mm2.cutesv.vcf HG00733.mm2.cutesv

Also called using svim:
svim alignment --min_sv_size 50 HG00733_linear_svim HG00733.linear.bam hg38.no_alts.fasta

svim alignment --min_sv_size 50 HG00733_mm2_svim HG00733.bam hg38.no_alts.fasta

truvari bench --chunksize 10000 -s 50 -r 2000 -P 0.5 -p 0 -b HG00733_dipcall.dip.tv-anno.norm.euch.sv.vcf.gz -c HG00733.linear.svim/HG00733.vcf.q10.euch.vcf.gz -o HG00733.linear.svim.S50.truvari -S 50 --includebed hg38.euchromatin.bed &

truvari bench --chunksize 10000 -s 50 -r 2000 -P 0.5 -p 0 -b HG00733_dipcall.dip.tv-anno.norm.euch.sv.vcf.gz -c HG00733.mm2.svim/HG00733.vcf.q10.euch.vcf.gz -o HG00733.mm2.svim.S50.truvari -S 50 --includebed hg38.euchromatin.bed &

truvari bench --chunksize 10000 -s 50 -r 2000 -P 0.5 -p 0 -b HG00733_dipcall.dip.tv-anno.norm.euch.sv.vcf.gz -c HG00733.linear.cutesv/HG00733.linear.cutesv.euch.vcf.gz -o HG00733.linear.cutesv.S50.truvari -S 50 --includebed hg38.euchromatin.bed &

truvari bench --chunksize 10000 -s 50 -r 2000 -P 0.5 -p 0 -b HG00733_dipcall.dip.tv-anno.norm.euch.sv.vcf.gz -c HG00733.mm2.cutesv/HG00733.mm2.cutesv.euch.vcf.gz -o HG00733.mm2.cutesv.S50.truvari -S 50 --includebed hg38.euchromatin.bed &


head */summary.json
==> HG00733.linear.cutesv.S50.truvari/summary.json <==
{
"TP-base": 12759,
"TP-comp": 12759,
"FP": 9998,
"FN": 16763,
"precision": 0.5606626532495496,
"recall": 0.4321861662488991,
"f1": 0.4881118613592456,
"base cnt": 29522,
"comp cnt": 22757,

==> HG00733.mm2.cutesv.S50.truvari/summary.json <==
{
"TP-base": 13687,
"TP-comp": 13687,
"FP": 2100,
"FN": 15835,
"precision": 0.8669791600684107,
"recall": 0.4636203509247341,
"f1": 0.6041625284159879,
"base cnt": 29522,
"comp cnt": 15787,

==> HG00733.linear.svim.S50.truvari/summary.json <==
{
"TP-base": 10618,
"TP-comp": 10618,
"FP": 3346,
"FN": 18904,
"precision": 0.7603838441707247,
"recall": 0.35966397940518935,
"f1": 0.4883410752885986,
"base cnt": 29522,
"comp cnt": 13964,

==> HG00733.mm2.svim.S50.truvari/summary.json <==
{
"TP-base": 14729,
"TP-comp": 14729,
"FP": 1867,
"FN": 14793,
"precision": 0.8875030127741624,
"recall": 0.498916062597385,
"f1": 0.6387527646472093,
"base cnt": 29522,
"comp cnt": 16596,

You can ignore the recall here because there may be variants found in the assemblies missing from the read based callsets. However, the number of true positives is dramatically lower for both algorithms, and false positives higher in both algorithms for linear compared to standard alignment.

I'm happy to give the rest of the feedback about the manuscript if it can be shown that linear can produce SV calls comparable or better than the alignment based calls.

**Authors’ response to reviewers**

**Contents**

0.1 Reviewer 2	2
0.2 Response 2	3
    0.2.1 Regarding how SVs are compared	3
    0.2.2 Regarding the meaning of supporting reads of an SV	3
    0.2.3 Regarding the assembly-based assessment	4

A Appendices	11

A.1 Assembly-based benchmark by reviewer 2	11
A.2 Assembly-based benchmark by the authors	12

## 0.1 Reviewer 2

The authors present a method to perform preprocessing of reads (filter) improve SV calling for long reads. In certain samples and conditions it is stated to improve metrics such as the F1 score.

Unfortunately, there are many critical issues with this study that prevent publication, at least in current form ranging from how the methods were evaluated to how the method is described: Evaluation: The methods used to evaluate are not adequate. In particular, there are truth sets that have had considerable effort, and methods developed to compare variant callsets. Genome in a bottle + Truvari is the gold standard, however other methods such as TT-Mars offer a

complementary approach.

Compare to Genome In A Bottle truth set (HG002). The number of variants per sample has a huge range, for example between 17774-28924 for HG002, for variants that are *>*= 80*bps*. Even the low number is likely an overestimate as most datasets have 22k calls for this individual starting at 50 bases, and the majority of SV are short.

At the least, describe how variants are compared (overlap? reciprocal overlap? etc.). What does it mean for a read to be supporting an SV?

I have many comments about the structure of the manuscript, and writing in general. How- ever, the review took a long time because I tried running a benchmark of linear and did not get positive results relative to simply running minimap2. One potential reason for this is that I used a gold standard variant callset (dipcall on a haplotype-resolved assembly), and only evaluated calls in the euchromatic regions of the genome. The trio analysis could suffer from problems of propagating errors in the parents and child.

I tried the following approach to evaluate linear: variants were called from the HPRC diploid assembly of HG00733 [https://github.com/human-pangenomics/HPP_Year1_Assemblies/blob/](https://github.com/human-pangenomics/HPP_Year1_Assemblies/blob/main/assembly_index/Year1_assemblies_v2_genbank.index) [main/assembly_index/Year1_assemblies_v2_genbank.index](https://github.com/human-pangenomics/HPP_Year1_Assemblies/blob/main/assembly_index/Year1_assemblies_v2_genbank.index)

The raw data for the assembly is available here: [https://s3-us-west-2.amazonaws.com/...](https://s3-us-west-2.amazonaws.com/human-pangenomics/index.html?prefix=submissions/8ced218e-9699-458e-9708-eef6969a8065--EXTRAMURAL_SAMPLES/HG00733/hifi/)

I called variants from hg38 using dipcall on the assembly. Assembly based SV calls have been shown to be extremely accurate, and a drop in for genome in a bottle truth set. The change from default was to use a different -z option: MM2 OPT=-xasm5 –cs -t$(N THREADS)

-z200000,10000

Only calls in the following regions were used:

| chr1 | 5000000 12170000 | 0 |
| --- | --- | --- |
| chr1 | 125100000 | 243956422 |
| ... |  |  |

head */ summary . json

== > HG 00733 . linear. cutesv . S50 . truvari/ summary . json <==

{

" TP - base ": 12759 ,

" TP - comp ": 12759 ,

" FP": 9998 ,

" FN": 16763 ,

" precision ": 0 .5606626532495496 ,

" recall": 0 .4321861662488991 ,

" f1 ": 0 .4881118613592456 ,

" base cnt": 29522 ,

" comp cnt": 22757 ,

}

== > HG 00733 . mm2 . cutesv . S50 . truvari/ summary . json <==

{

" TP - base ": 13687 ,

" TP - comp ": 13687 ,

" FP": 2100 ,

" FN": 15835 ,

" precision ": 0 .8669791600684107 ,

" recall": 0 .4636203509247341 ,

" f1 ": 0 .6041625284159879 ,

" base cnt": 29522 ,

" comp cnt": 15787 ,

}

== > HG 00733 . linear. svim . S50 . truvari/ summary . json <==

{

" TP - base ": 10618 ,

" TP - comp ": 10618 ,

" FP": 3346 ,

" FN": 18904 ,

" precision ": 0 .7603838441707247 ,

" recall": 0 .35966397940518935 ,

" f1 ": 0 .4883410752885986 ,

" base cnt": 29522 ,

" comp cnt": 13964 ,

}

== > HG 00733 . mm2 . svim . S50 . truvari/ summary . json <==

{

" TP - base ": 14729 ,

" TP - comp ": 14729 ,

" FP": 1867 ,

" FN": 14793 ,

" precision ": 0 .8875030127741624 ,

" recall": 0 .498916062597385 ,

" f1 ": 0 .6387527646472093 ,

" base cnt": 29522 ,

" comp cnt": 16596 ,

}

You can ignore the recall here because there may be variants found in the assemblies missing from the read based callsets. However, the number of true positives is dramatically lower for both algorithms, and false positives higher in both algorithms for linear compared to standard alignment.

I’m happy to give the rest of the feedback about the manuscript if it can be shown that linear can produce SV calls comparable or better than the alignment based calls.

## 0.2 Response 2

We thank the reviewer very much for reviewing the manuscript, conducting the evaluation with detailed descriptions and pointing out the issues based on the results. Our responses to the issues are as follows:

### 0.2.1 Regarding how SVs are compared

We have added the following description in the manuscript section 2.1: *“ Two SVs are compared using reciprocal overlap and are considered to be identical if a proportion of their individual sizes overlap and their genotypes are matched. For insertions and duplications, which have no physical span over the reference, we compare its virtual reference span, defined as a span starting at the SV position and ending at the position an SV length away from the starting position.”*

### 0.2.2 Regarding the meaning of supporting reads of an SV

In the manuscript, supporting reads of an SV refer to reads containing the SV signatures, which are pieces of evidence of the SV. The word “coverage” is also used in the manuscript as the number of supporting reads for simplicity. cuteSV and SVIM use the keywords “RE” and “SUPPORT” in the VCF file to specify the number of supporting reads. We extract the keywords “RE” and “SUPPORT” in outputs as the number of supporting reads in the assessments.

### 0.2.3 Regarding the assembly-based assessment

Thank you for pointing out that the assembly-based assessment should be employed to im- prove the assessment quality. We have conducted the assembly-based assessment with the main steps in Appendix.2 based on the reviewer’s assessment in Appendix A.1 and complemented the manuscript with the assembly-based assessment in Section 2.3.

Regarding the false positives (precision), it is worth noting that the Filter is developed to complement the detectable SV space of aligners. Thus it is designed to filter as many SV signatures as possible, especially nonlinear intra-read SVs that are probably missed by aligners. Inevitably, it is more likely to report more false positives. Figure 1 of the trio-based assessment reprinted from the manuscript shows that aligners have higher precision in detecting insertions and deletions especially for lower supporting reads. However, false positives like short insertions and deletions can be eliminated by post-processing such as additional local alignment of SVs. The Filter can be used as a preprocessor. In the Results section of the manuscript:

*“It is a standalone tool compatible with SV callers and a preprocessor that can improve the performance of existing aligner-based pipelines for long-read SV detection”*.

We tried to show the strategy, in the manuscript Figure 1, where the Filter is connected to the Aligner with a dashed arrow.

Therefore, in the following discussion, we focus on the difference between the true positives (recall) of the two assessments. The differences are largely attributable to the arguments used by the Filter-based pipelines. The results show that the two assessments are essentially consistent. It is also worth noting that the assembly-based true SVs set are largely based on minimap2. Specifically, the following ***issues*** may cause assessment biases.

**Issues regarding the assembly-based true SV set**

1. Study [SVIM-asm](https://academic.oup.com/bioinformatics/article/36/22-23/5519/6042701), which is another tool for assembly-based SV calls, evaluated the recall and precision in two diploid assemblies, shown as Asm A and Asm B in Figure 2. In the second row of the figure, the precision and recall of Dipcall are approximately 80% and 85%. Thus, precisions and recalls are possibly biased as well when it is used as the true set.
2. Dipcall methods show that it uses minimap2 to align the assembly to the reference genomes and call SVs by comparing the assembly-genome alignment. Documentations of the projects show that both [HPRC assembly](https://github.com/human-pangenomics/HPP_Year1_Assemblies/tree/main) and [HG00733 reads](https://s3-us-west-2.amazonaws.com/human-pangenomics/index.html?prefix=submissions/8ced218e-9699-458e-9708-eef6969a8065--EXTRAMURAL_SAMPLES/HG00733/hifi/) are based on PacBio HiFi reads. Thus using Dipcall to compute the HPRC assembly-genome alignment is essentially very like using minimap2 to compute the HG00733 read-genome alignment.

The documentation also shows that the assembly is assembled by using hifiasm. hifiasm and minimap2 may share common algorithms, since they are developed by the same team. Both hifiasm and minimap2 are great. However, the problem is that propagation of uncertainty is more likely to happen when using data generated by hifiasm to evaluate minimap2.

Thus the results may be biased toward minimap2 to some extent.

**Raw results** Recalls and precisions of Filter- and aligner-SV callers for each level of supporting reads are shown in Figure 3. It is measured by Truvari with the same arguments in the reviewer’s

HG002.cuteSV	HG005.cuteSV	HG002.SVIM	HG005.SVIM

1.0

0.8

0.6

Precision

0.4

0.2

0.0

1.0

Aligner Filter

0.8

0.6

Recall

0.4

0.2

0.0

2 4 6 8 10 12 14 16	2 4 6 8 10 12 14 16	2 4 6 8 10 12 14 16	2 4 6 8 10 12 14 16

coverage

**Figure 1:** Trio-based assessment precision and recall for 4 pipelines on dataset HG002, HG005. Reprinted from the manuscript.

assessment. We employed the additional ***NGMLR-based pipelines***, which is another accurate long-read aligner, as the control of possible biases. We observed that the differences in perfor- mances are largely attributable to the false positives shown as ’FP’ in the following example of Truvari results, which is for 6 supporting reads and minimum SV length 100bps.

Filter+ cuteSV	minimap 2 + cuteSV

" TP - base ": 9475 ,	" TP - base ": 9322 ,

" TP - call ": 9475 ,	" TP - call ": 9322 ,

" FP ": 5840 ,	" FP ": 2628 ,

" FN ": 3863 ,	" FN ": 4016 ,

" precision ": 0 .6186745021221025 ,	" precision ": 0 .7800836820083682 ,

" recall ": 0 .7103763682711051 ,	" recall ": 0 .6989053831159094 ,

The false positives of Filter-based pipelines are probably ***overestimated*** due to the underlying biases discussed above. A proportion of false positives is possibly true positives but is not recorded in the true SV set. Correspondingly, true positives in the following expression are underestimated since SVs detected by the Filter are constant. In such a case, both the precision and recall in the following expression are probably underestimated.

*false positives* + *true positives* = *SV s detected by Filter*

*precision* =

*recall* =

*true positives*

*true positives* + *false positives true positives*

*true positives* + *false negatives*

(1)

**Compared to the trio-based assessment** We then compared the trio-based assessment to the assembly-based assessment to see if the two assessments are effective. The assembly-based and trio-based assessments define the true SV set differently. However, ***the results of the two assessments are essentially consistent that***

*recallFilter|Aligner ≈* 1

**Figure 2:** Recall and precision of Dipcall and SVIM-asm. Reprinted from the [SVIM-asm study](https://academic.oup.com/bioinformatics/article/36/22-23/5519/6042701).

Namely, most true SVs detected by the Aligner can be detected by the Filter. The reasons are as follows:

1. The recall for SV sets *X* and *Y* in the manuscript is defined as,

*recall_X|Y_* =

*|X ∩ Y |*

=

*|Y |*

SVs in *X* and *Y*

SVs in *Y*

Then the statistical recall in expression 1 for Filter denoted by *Recall_F_ _ilter_* is equal to

True SVs detected by Filter

*Recall_F_ _ilter_* =

True SVs	= *recallFilter|True SV s*

Strictly speaking, the true SV sets used in the assembly-based and trio-based assessments are approximations of the true ones. In the trio-based assessment, true SVs are defined as high-confidence SVs which are SVs mainly detected by cuteSV-based pipelines (since cuteSV detects most SVs detected by SVIM) with *≥* 10 supporting reads and validated by Mendelian inheritance. For simplicity, we denote the true SV set of trios as follows,

True SVs of tiros *≈* High-confidence SVs by Filter-cuteSV L Aligner-cuteSV

= Filter L Aligner

Thus, the recall of Filter in the trio assessment is

*RecallFilter* = *recallFilter|True SV s*

= *recallFilter|Filter∪Aligner*

*≤ recallFilter|Aligner*

Since most true SVs of trios are detected by cuteSV, we have

*RecallFilter* = *RecallFiter*-*cuteSV ∪Filter*-SVIM *≈ RecallFilter*-*cuteSV*

And we can find in figure 1 that *Recall_F_ _ilter−cuteSV_ ≈* 1, especially for lower supporting reads. Therefore,

*recallFilter|Aligner ≥ RecallFilter ≈* 1

For the assembly-based assessment, *recall_F_ _ilter|Aligner_* is computed by comparing true pos- itive files generated by Truvari named tp-base.vcf. It is computed as follows.

True SVs detected by Filter and Aligner

*recallFilter|Aligner* =

True SVs detected by Aligner

Identical records in tp-base.vcf files of Filter and Aligner

=

Records in tp-base.vcf of Aligner

Table 1 shows *recall_F_ _ilter|Aligner_* of assembly-based assessment. We can find that it is approximately equals to 1, especially for lower supporting reads as well. Therefore, trio- based assessment and aligner-based assessment are consistent in terms of the recall.

**Table 1:** *recallFilter−cuteSV |Aligner−cuteSV* and *recallFilter−SV IM|Aligner−SV IM* versus supporting reads in the assembly-based assessment.

| *recall* | 2 | 4 | 6 | 8 | 10 | 12 | 14 | 16 |
| --- | --- | --- | --- | --- | --- | --- | --- | --- |
| cuteSV | 0.990861 | 0.968392 | 0.95516 | 0.943326 | 0.931093 | 0.917259 | 0.905167 | 0.888142 |
| SVIM | 0.998574 | 0.94877 | 0.913454 | 0.889538 | 0.868488 | 0.846652 | 0.837556 | 0.819277 |

2. On the other hand, we put Figure 3 and Figure 1 together in Figure 4 to compare the

*Recall* in expression 1 for the two assessments.

First, the shapes of the curves of the two assessments are similar.

Second, we shift all blue-dashed curves upwards by ∆ to amend the possible biases. Then the distances between dashed curves (assembly) are approximately equals to those between the solid curves (trio). It can be expressed as,

*yassembly,Filter* (*x*) *− yassembly,aligner* (*x*) + ∆ *≈ ytrio,Filter* (*x*) *− ytrio,aligner* (*x*)

where ∆ is for bias correction, *x* is the coverage and *y* is the recall or precision in the figure. The expression holds for subfigure HG002.cuteSV and HG005.cuteSV when *x >* 6 and other subfigures when *x >* 0.

Therefore, we think the two assessments are consistent. The amended recall and precision are likely between those of the two assessments.

**Compared to the SV space assessment** The results of the assembly-based assessment are ***consistent*** with the SV space assessment as well. Figure 5 is the SV space assessment results reprinted from the manuscript. We observed that minimap2 performs ***better*** in detecting ***in- sertions and deletions***. The recall of Filter in detecting insertions and deletions in reads of average sequencing error=0.1 in the figure, which is relatively closer to the sequencing error of HiFi reads, is comparable to minimap2. The precision of NGMLR in detecting insertions and deletions is the highest. The results are consistent with the assembly-based assessment, which evaluated the performances of detecting insertions and deletions in HiFi reads. In contrast, the

HG00733.cuteSV	HG00733.SVIM

1.0

minimap2 Filter NGMLR

0.8

0.6

Precision

0.4

0.2

0.0

1.0

0.8

0.6

Recall

0.4

0.2

0.0

2	4	6	8	10	12	14	16	2	4	6	8	10	12	14	16

coverage

**Figure 3:** Assembly-based assessment precision and recall for 6 pipelines on dataset HG00733. The horizontal axis of coverage is the minimum number of reads to call an SV.

Filter and NGMLR perform ***better*** in detecting nonlinear SVs including the ***inversions and duplications***, since the Filter is developed to complement aligner-based pipelines, to detect nonlinear intra-read SVs. The results show that Aligners and Filter have its advantages and lim- itations. Currently, the Filter is much ***faster*** than the aligners to compute (I/O time excluded). Therefore, we think the Filter still has much ***potential to optimize*** for efficient detection of more diverse SVs.

**Issues of combining the Filter with SV callers** SV callers taking SAM/BAM as input regard Filter results as alignment, though the Filter results are not alignment. Since SV callers are developed for alignments, it inevitably limits the Filter-based pipeline performance. We have discussed the problem and potential solutions in the Discussion of the manuscript:

“*We assessed the divergence of detected endpoints for simulated reads and SVs, as shown in Figure 5. Endpoints of SV signatures reported by the Filter are commonly* ***more divergent*** *than those reported by the aligner.* ***As a result, SV callers can possibly fail to compute the common endpoints for SV signatures.*** *Consequently, 2-4 additional supporting reads are required for Filter-based pipelines to achieve comparable precision, as shown in Figure 3. This is one of the reasons why the precision of the Filter-based pipelines is lower than the precision of the aligner-based pipelines in the trio-based assessment. We found that a considerable number of SVs, which are not correctly detected by SV callers, are detected by the Filter. We simply visual- ized these SVs by using IGV and found that despite the significant divergence, detected endpoints in different supporting reads were distributed around the true endpoint, clearly indicating the existence of the common SV. Thus, a considerable number of SVs missed by SV callers are due to the divergence of the endpoints that SV callers cannot process*

We mentioned two solutions to further improve the performance in the future: “*We have at least two solutions to further improve the performance of Filter-based pipelines. The first is to* ***1.implement a very lightweight alignment module in the Filter to align a short subsequence containing endpoints for each detected SV signature.*** *It can significantly*

1.0

0.8

0.6

0.4

0.2

0.0

1.0

0.8

0.6

0.4

0.2

0.0

HG002.cuteSV	HG005.cuteSV	HG002.SVIM	HG005.SVIM

Trio−based Assembly−based

minimap2 Filter

2 4 6 8 10 12 14 16	2 4 6 8 10 12 14 16	2 4 6 8 10 12 14 16	2 4 6 8 10 12 14 16

Precision

Recall

coverage

**Figure 4:** Comparison of the assembly-based and trio-bases assessment results by putting together curves in figure 3 shown as dashed curves and curves in figure 1 shown as solid curves.

*reduce the divergence of endpoints in supporting reads. Then, SV callers are more likely to com- pute the common endpoints for supporting reads. It is easier to implement and is more compatible with existing SV callers. The second solution is to* ***2.develop a brand new SV caller with optimized models and parameters for the Filter****. Although it is more sophisticated to im- plement, the potential of the Filter-based pipeline can possibly be fully tapped.* ”

**INS/DEL**

**INV**

**DUP**

(2k,0.1) (0.1k,0.2) (0.1k,0.15)

(2k,0.1) (0.1k,0.2) (0.1k,0.15)

(2k,0.1) (0.1k,0.2) (0.1k,0.15)

| (2k,0.15)  (2k,0.2)  (1.5k,0.1) | 100%  50% | (0.1k,0.1)  (0.2k,0.2)  (0.2k,0.15) | (2k,0.15)  (2k,0.2)  (1.5k,0.1) | 100%  50% | (0.1k,0.1)  (0.2k,0.2)  (0.2k,0.15) | (2k,0.15)  (2k,0.2)  (1.5k,0.1) | 100%  50% | (0.1k,0.1)  (0.2k,0.2)  (0.2k,0.15) |
| --- | --- | --- | --- | --- | --- | --- | --- | --- |
| (1.5k,0.15) |  | (0.2k,0.1) | (1.5k,0.15) |  | (0.2k,0.1) | (1.5k,0.15) |  | (0.2k,0.1) |
| (1.5k,0.2) | 0% | (0.3k,0.2) | (1.5k,0.2) | 0% | (0.3k,0.2) | (1.5k,0.2) | 0% | (0.3k,0.2) |
| (1k,0.1) |  | (0.3k,0.15) | (1k,0.1) |  | (0.3k,0.15) | (1k,0.1) |  | (0.3k,0.15) |
| (1k,0.15) |  | (0.3k,0.1) | (1k,0.15) |  | (0.3k,0.1) | (1k,0.15) |  | (0.3k,0.1) |
| (1k,0.2) |  | (0.4k,0.2) | (1k,0.2) |  | (0.4k,0.2) | (1k,0.2) |  | (0.4k,0.2) |
| (0.5k,0.1) |  | (0.4k,0.15) | (0.5k,0.1) |  | (0.4k,0.15) | (0.5k,0.1) |  | (0.4k,0.15) |

(0.5k,0.15) (0.5k,0.2) (0.4k,0.1)

Filter precision Filter recall

(2k,0.1) (0.1k,0.2) (0.1k,0.15)

(0.5k,0.15) (0.5k,0.2) (0.4k,0.1)

Filter precision Filter recall

(2k,0.1) (0.1k,0.2) (0.1k,0.15)

(0.5k,0.15) (0.5k,0.2) (0.4k,0.1)

Filter precision Filter recall

(2k,0.1) (0.1k,0.2) (0.1k,0.15)

| (2k,0.15)  (2k,0.2)  (1.5k,0.1) | 100%  50% | (0.1k,0.1)  (0.2k,0.2)  (0.2k,0.15) | (2k,0.15)  (2k,0.2)  (1.5k,0.1) | 100%  50% | (0.1k,0.1)  (0.2k,0.2)  (0.2k,0.15) | (2k,0.15)  (2k,0.2)  (1.5k,0.1) | 100%  50% | (0.1k,0.1)  (0.2k,0.2)  (0.2k,0.15) |
| --- | --- | --- | --- | --- | --- | --- | --- | --- |
| (1.5k,0.15) |  | (0.2k,0.1) | (1.5k,0.15) |  | (0.2k,0.1) | (1.5k,0.15) |  | (0.2k,0.1) |
| (1.5k,0.2) | 0% | (0.3k,0.2) | (1.5k,0.2) | 0% | (0.3k,0.2) | (1.5k,0.2) | 0% | (0.3k,0.2) |
| (1k,0.1) |  | (0.3k,0.15) | (1k,0.1) |  | (0.3k,0.15) | (1k,0.1) |  | (0.3k,0.15) |
| (1k,0.15) |  | (0.3k,0.1) | (1k,0.15) |  | (0.3k,0.1) | (1k,0.15) |  | (0.3k,0.1) |
| (1k,0.2) |  | (0.4k,0.2) | (1k,0.2) |  | (0.4k,0.2) | (1k,0.2) |  | (0.4k,0.2) |
| (0.5k,0.1) |  | (0.4k,0.15) | (0.5k,0.1) |  | (0.4k,0.15) | (0.5k,0.1) |  | (0.4k,0.15) |

(0.5k,0.15) (0.5k,0.2) (0.4k,0.1)

Minimap2 precision Minimap2 recall

(2k,0.1) (0.1k,0.2) (0.1k,0.15)

(0.5k,0.15) (0.5k,0.2) (0.4k,0.1)

Minimap2 precision Minimap2 recall

(2k,0.1) (0.1k,0.2) (0.1k,0.15)

(0.5k,0.15) (0.5k,0.2) (0.4k,0.1)

Minimap2 precision Minimap2 recall

(2k,0.1) (0.1k,0.2) (0.1k,0.15)

| (2k,0.15)  (2k,0.2)  (1.5k,0.1) | 100%  50% | (0.1k,0.1)  (0.2k,0.2)  (0.2k,0.15) | (2k,0.15)  (2k,0.2)  (1.5k,0.1) | 100%  50% | (0.1k,0.1)  (0.2k,0.2)  (0.2k,0.15) | (2k,0.15)  (2k,0.2)  (1.5k,0.1) | 100%  50% | (0.1k,0.1)  (0.2k,0.2)  (0.2k,0.15) |
| --- | --- | --- | --- | --- | --- | --- | --- | --- |
| (1.5k,0.15) |  | (0.2k,0.1) | (1.5k,0.15) |  | (0.2k,0.1) | (1.5k,0.15) |  | (0.2k,0.1) |
| (1.5k,0.2) | 0% | (0.3k,0.2) | (1.5k,0.2) | 0% | (0.3k,0.2) | (1.5k,0.2) | 0% | (0.3k,0.2) |
| (1k,0.1) |  | (0.3k,0.15) | (1k,0.1) |  | (0.3k,0.15) | (1k,0.1) |  | (0.3k,0.15) |
| (1k,0.15) |  | (0.3k,0.1) | (1k,0.15) |  | (0.3k,0.1) | (1k,0.15) |  | (0.3k,0.1) |
| (1k,0.2) |  | (0.4k,0.2) | (1k,0.2) |  | (0.4k,0.2) | (1k,0.2) |  | (0.4k,0.2) |
| (0.5k,0.1) |  | (0.4k,0.15) | (0.5k,0.1) |  | (0.4k,0.15) | (0.5k,0.1) |  | (0.4k,0.15) |
| (0.5k,0.15) (0.5k,0.2) (0.4k,0.1) | | | (0.5k,0.15) (0.5k,0.2) (0.4k,0.1) | | | (0.5k,0.15) (0.5k,0.2) (0.4k,0.1) | | |

NGMLR precision NGMLR recall

NGMLR precision NGMLR recall

NGMLR precision NGMLR recall

**Figure 5:** The detectable SV space of aligners and the Filter measured by the recall and precision of detecting different types of SVs. Values in each figure are labeled by lengths(bps) of the SVs and the sequencing error on average. Reprinted from the manuscript.

# A Appendices

## A.1 Assembly-based benchmark by reviewer 2

| chr1 | 5000000 12170000 | 0 |
| --- | --- | --- |
| chr1 | 125100000 | 243956422 |
| ... |  |  |
| chrX | 63800000 | 151040895 |
| chrY | 5000000 10300000 |  |
| chrY | 10600000 | 52227415 |

truvari anno svinfo HG 00733 _dipcall . dip . vcf. gz | bgzip -c > HG 00733 _dipcall . dip . tv

*'→* - anno . vcf. gz

bcftools norm -m -	HG 00733 _dipcall . dip . tv - anno . vcf. gz	> HG 00733 _dipcall . dip . tv

*'→* - anno . norm . vcf. gz

bedtools intersect - header -a HG 00733 _dipcall . dip . tv - anno . norm . vcf. gz -b hg38 .

*'→* euchromatin . bed -u | bgzip -c	> HG 00733 _dipcall . dip . tv - anno . norm . euch . vcf.

*'→* gz

Next , reads were aligned using minimap 2 and preprocessed with linear.

zcat $DIRC / projects/ HPRC / Assemblies_v 1 / HG 00733 /*. gz	| minimap 2 $hg38 na / hg38 .

*'→* no_alts. fasta - -x map - hifi -a -t 30 | samtools sort - m6G -@2 -o HG 00733 .

*'→* bam

samtools index -@2 HG 00733 . bam #!/ usr/ bin / env bash

linear	$DIRC / projects/ HPRC / Assemblies_v 1 / HG 00733 /*. gz x $hg38 na / hg38 . no_alts.

*'→* fasta	- ot 3 -o HG 00733 . linear - rg hgsvg - sn " HG 00733 " -t 24

Variants were called using cutesv :

#!/ usr/ bin / env bash

cuteSV -- genotype -- max_cluster_bias_INS 1000 -- diff_ratio_merging_INS 0.9	-- *'→* diff_ratio_merging_DEL 0.5	-t 8	HG 00733 . linear. bam	hg38 . no_alts. fasta *'→* HG 00733 . linear. cutesv . vcf HG 00733 . linear. cutesv

#!/ usr/ bin / env bash conda activate

cuteSV -- genotype -- max_cluster_bias_INS 1000 -- diff_ratio_merging_INS 0.9	--

*'→* diff_ratio_merging_DEL 0.5	-t 8	./ HG 00733 . bam	hg38 . no_alts. fasta

*'→* HG 00733 . mm2 . cutesv . vcf HG 00733 . mm2 . cutesv Also called using svim :

svim alignment -- min_sv_size 50 HG 00733 _linear_svim HG 00733 . linear. bam	hg38 .

*'→* no_alts. fasta

svim alignment -- min_sv_size 50 HG 00733 _mm 2 _svim HG 00733 . bam	hg38 . no_alts. fasta truvari bench -- chunksize 10000 -s 50	-r 2000 -P 0.5 -p 0 -b HG 00733 _dipcall . dip .

*'→* tv - anno . norm . euch . sv. vcf. gz	-c HG 00733 . linear. svim / HG 00733 . vcf. q10 . euch .

*'→* vcf. gz -o HG 00733 . linear. svim . S50 . truvari -S 50 -- includebed hg38 .

*'→* euchromatin . bed	&

truvari bench	-- chunksize 10000 -s 50 -r 2000 -P 0.5 -p 0 -b HG 00733 _dipcall . dip . *'→* tv - anno . norm . euch . sv. vcf. gz	-c HG 00733 . mm2 . svim / HG 00733 . vcf. q10 . euch . vcf. *'→* gz -o HG 00733 . mm2 . svim . S50 . truvari	-S 50 -- includebed hg38 . euchromatin . bed *'→*	&

truvari bench	-- chunksize 10000 -s 50 -r 2000 -P 0.5 -p 0 -b HG 00733 _dipcall . dip . *'→* tv - anno . norm . euch . sv. vcf. gz	-c HG 00733 . linear. cutesv / HG 00733 . linear. cutesv *'→* . euch . vcf. gz -o HG 00733 . linear. cutesv . S50 . truvari -S 50 -- includebed hg38 . *'→* euchromatin . bed &

truvari bench -- chunksize 10000 -s 50 -r 2000 -P 0.5 -p 0 -b HG 00733 _dipcall . dip . *'→* tv - anno . norm . euch . sv. vcf. gz -c HG 00733 . mm2 . cutesv / HG 00733 . mm2 . cutesv . euch . *'→* vcf. gz -o HG 00733 . mm2 . cutesv . S50 . truvari -S 50 -- includebed hg38 .

*'→* euchromatin . bed &

## A.2 Assembly-based benchmark by the authors

\# >Minimap2 - based pipeline :

\# Run minimap2 - SV caller and using the review ’ s arguments as shown in Appendix .1

\# >Filter - based pipeline :

\# Main differences compared to Appendix .1:

\#	Run filter with preset arguments -p

\#	Run cuteSV and SVIM with default arguments for Filter HiFi reads #	Run truvari with -s 100 for Aligners and Filter.

\# 1. Update to the latest version .

git clone https :// github . com / xp3i4 / linear cmake && make

\# 2. run the filter with argument -p 2 to enable the second group of parameters for *'→* cuteSV or SVIM with HiFi reads. We also suggest to use -p 1 for cuteSV for *'→* better precisions .	We may use different running parameters for different *'→* SV callers since SV callers are currently optimized for alignments instead *'→* of Filter results.

linear filter $DIR / HG 00733 * gz x hg38 . fa -o HG 00733 _filter -p 2 -t 8	# for SVIM and

*'→*	cuteSV

linear filter $DIR / HG 00733 * gz x hg38 . fa -o HG 00733 _filter -p 1 -t 8	# optional , to

*'→*	better integrate cuteSV

\# 3. Convert sam to bam and index

samtools sort HG 00733 _filter . sam -O bam -o HG 00733 _filter_sort . bam samtools index HG 00733 _filter_sort . bam

\# 4. run SV callers with default parameters

\# It is worth noting that arguments of SV callers for Filter results are different

*'→* from those for Alignments. Arguments of cuteSV optimized for HiFi read *'→* alignment shown as follows are not suitable for Filter results. Because *'→* Filter results are not alignments , though they are in SAM / BAM format. *'→* Currently , we suggest to use default arguments.

\#	cuteSV -h

\#	For PacBio CCS( HIFI) data :

\#	-- max_cluster_bias_INS	1000

\#	-- diff_ratio_merging_INS	0.9

\#	-- max_cluster_bias_DEL	1000

\#	-- diff_ratio_merging_DEL	0.5

\# Argument of cuteSV -s 2 is to call SVs with supporting reads >=2 , since we need

*'→* to group SVs by supporting reads from 2 to 16 in the following steps. It

*'→* will take longer time for cuteSV to run with -s	2

cuteSV -- genotype -s 2 HG 00733 _filter_sort . bam hg38 . fa HG 00733 _filter_cute SV . vcf

*'→* tmp

svim alignment HG 00733 _filter_sort . bam hg38 . fa

\# 5. Extract INS and DEL since the true set comprises insertions and deletions.

grep -e "#" -e INS -e DEL HG 00733 _filter_cute SV . vcf > HG 00733 _filter_cute SV_indels

*'→* . vcf

grep -e "#" -e INS -e DEL HG 00733 _filter_svim . vcf > HG 00733 _filter_svim_indels . vcf

\# 6. Extract SVs whose supporting reads >= 6 ( key : RE for cuteSV results and SUPPORT

*'→*	for SVIM results)

\# parse Vcf. awk is a simple script to extract SVs whose supporting reads >=

*'→* argument.

\# We run the script to extract supporting reads from 2 to 16 for the recall and

*'→* precision Figure in the manuscript .

parse Vcf. awk	HG 00733 _filter_cute SV_indels . vcf " RE=" 6 >

*'→* HG 00733 _filter_cute SV_indels_6 . vcf

parse Vcf. awk	HG 00733 _filter_svim_indels . vcf " SUPPORT =" 6 >

*'→* HG 00733 _filter_svim_indels_6 . vcf

\# 7 Use bgzip and tabix to process vcf file bgzip HG 00733 _filter_cute SV_indels_6 . vcf bgzip HG 00733 _filter_svim_indels_6 . vcf

tabix -p HG 00733 _filter_cute SV_indels_6 . vcf. gz tabix -p HG 00733 _filter_svim_indels_6 . vcf. gz

\# 8. Run truvari with the arguments used by the reviewer except two arguments.

\# -- chunksize 10000 are removed since it seems not available in version v3 .0.0.

#-s 50 are replaced by -s 100 , namely the minimum size of SV length is set to 100 *'→* bps. Since the Filter is for >100 bp SVs. All assessments in the manuscript *'→* are based on SVs >= 100 bps.

truvari bench -s 100 -r 2000 -P 0.5 -p 0 -b HG 00733 _dipcall . dip . tv - anno . norm . euch .

*'→* vcf. gz	-c HG 00733 _filter_cute SV_indels_6 . vcf. gz -o

*'→* HG 00733 _filter_cute SV_indels_6 . tr -S 50 -- includebed hg38 . euchromatin . bed truvari bench -s 100 -r 2000 -P 0.5 -p 0 -b HG 00733 _dipcall . dip . tv - anno . norm . euch .

*'→* vcf. gz	-c HG 00733 _filter_svim_indels_6 . vcf. gz -o

*'→* HG 00733 _filter_svim_indels_6 . tr -S 50 -- includebed hg38 . euchromatin . bed

**Second round of review**

**Reviewer 1**

There have been significant changes made to the manuscript. Chief among them for me is the improvement to the readability. As well as a new benchmark using an high quality assembly based truth set for HG00733 from the Human Pangenome Reference Consortium. Thank you to the authors for putting in this effort. With the improved readability, I have gone through the manuscript in greater detail. Before I comment on new items, I will briefly touch on the response to my first review.

Notes on responses
1. Compilation Error on Mac: Understood. It's a reasonable requirement.

2. Data Sources for training: It is good that data sources (real and simulated) were described. I would still like to know how data was separated for training/validation/testing. My main question here is whether the AAE model was trained/tested on the whole genome. Deep learning models are powerful enough to even memorize randomly labelled data. There is a high genomic similarity between individuals. Even when not training on sequence directly, the data can contain spurious statistical artifacts that a deep learning model can rely on to make predictions (ie the model could overfit on these details). It can be hard to detect when a model overfits when the training/testing data has too much unintended overlap.
The final model can certainly be trained on the whole genome, but there should be an experiment done where you train on only part of the genome and test on the held out portion. This can give a better indication on whether the model can truly generalize to unseen data.

3. AAE details moved to supplement: That is a good decision and the added paragraph does well enough describe the purpose of this stage of the pipeline.

4. Justification for the usage of "Filter": I understand your justification, but I don't quite agree with the blanket usage of the term filter. I suggest that you do the following to make it easier to understand your intention. For example in the abstract we could say: "Here, we present a new type of pipeline for population-scale long-read SV detection, the filter-based pipeline." Repeat this when introducing the pipeline in the rest of the manuscript as well.
Then, you must then make some sort of distinction when referring to the idea of a "filter based pipeline" and your filter based pipeline. Simply capitalising the word "filter" doesn't really disambiguate the two concepts enough.

For example in line 36, page 2 you say that Filter based pipelines use generative models to generate most likely results, but you really mean that your pipeline does that. Other "filter based" pipelines in the future may do something completely different to solve the same problem. Making general statements about the utility of "filter based" pipelines don't make really sense. We are only evaluating the utility of your "filter based" pipeline. You should just use the name of the tool (Linear) when referring to it.

New Items
Notes on the added assembly analysis:
-"However, a proportion of false positives are likely to be true positive SVs but are missed by the Dipcall. The real recall and precision of the Filter-based pipelines are actually higher than the evaluated ones."
--This sounds like speculation to me. I'm sure there are some missed true positives, but you need to connect the dots between these statements and the quantification of the drop in precision and recall. You do mention the idea that dipcall could be biased towards minimap2 based benchmarks, and present using an NGMLR based pipeline as a control for this bias, but once again, there needs to be a more quantified analysis for me to take those statements as fact.
-Also, it seems to me like there is a pretty unfavorable trade off as you increase the evidence threshold. The Alignment based methods have pretty decent precision/recall with low evidence coverage. The filter method, on the other hand, starts with atrocious precision, and rises to the level of "OK" (at least for SVIM) as you add evidence. But the recall drops to unacceptable levels before precision even approaches something decent. Please let me know if I am misinterpreting the precision/recall numbers here or if I am missing a key piece of information that helps put this observation into perspective.

Notes on the computational time analysis
-With the above notes in mind, it seems like the main advantage of the Filter based pipeline presented is to provide a preprocessing step that could drastically speed up the SV calling process without hurting precision/recall too much.
-You perform benchmarks using x1 up to x16 threads. From these benchmarks we begin to see diminishing returns from the Filter pipeline, but the benefits are still present at x16 threads.
--Here is my problem with this array of benchmarks. Modern computing clusters will have far more resources available than just x16 threads. I would like to see the analysis extended to x64 or even x128 threads.
---Will the main advantange of the Filter based pipeline disappear?
---While it is true that the memory useage will increase it will still be far less than what many computing clusters have at their disposal. My laptop is on par with the current benchmarks.

Conclusion
In my mind, the tradeoff between loss of precision/recall on a high quality assembly based benchmark and the vanishing computational benefits with modern hardware leads me to believe that this method may not be as usefull as initially thought. I still think that using a filter for reads before alignment/SV calling is a interesting idea, but as you mentioned in the discussion -- perhaps the benefits of the filter can be maximized if a SV caller is tuned specifically for the filter, or a new SV caller is built around the filter itself.

**Reviewer 2**

The authors have addressed my comments.

**Authors’ response to reviewers**

1 Reviewer: 1	2
  1.1 First review	2
  1.2 New items of notes on the added assembly analysis:	3
  1.3 New items of notes on the computational time analysis	5
  1.4 Conclusion	6

# 1 Reviewer: 1

## 1.1 First review

There have been significant changes made to the manuscript. Chief among them for me is the improvement to the readability. As well as a new benchmark using a high-quality assembly-based truth set for HG00733 from the Human Pangenome Reference Consortium. Thank you to the authors for putting in this effort. With the improved readability, I have gone through the manuscript in greater detail. Before I comment on new items, I will briefly touch on the response to my first review.

1. Compilation Error on Mac: Understood. It’s a reasonable requirement.
2. Data Sources for training: It is good that data sources (real and simulated) were described. I would still like to know how data was separated for training/validation/testing. My main question here is whether the AAE model was trained/tested on the whole genome. Deep learning models are powerful enough to even memorize randomly labeled data. There is a high genomic similarity between individuals. Even when not training on sequence directly, the data can contain spurious statistical artifacts that a deep learning model can rely on to make predictions (i.e. the model could overfit on these details). It can be hard to detect when a model overfits when the training/testing data has too much unintended overlap. The final model can certainly be trained on the whole genome, but there should be an experiment done where you train on only part of the genome and test on the held-out portion. This can give a better indication of whether the model can truly generalize to unseen data.
3. AAE details moved to supplement: That is a good decision and the added paragraph does well enough to describe the purpose of this stage of the pipeline.
4. Justification for the usage of "Filter": I understand your justification, but I don’t quite agree with the blanket usage of the term filter. I suggest that you do the following to make it easier to understand your intention. For example, in the abstract, we could say: "Here, we present a new type of pipeline for population-scale long-read SV detection, the filter-based pipeline." Repeat this when introducing the pipeline in the rest of the manuscript as well. Then, you must then make some sort of distinction when referring to the idea of a "filter-based pipeline" and your filter-based pipeline. Simply capitalizing the word "filter" doesn’t really disambiguate the two concepts enough.

For example, in line 36, page 2 you say that Filter-based pipelines use generative models to generate the most likely results, but you really mean that your pipeline does that. Other "filter-based" pipelines in the future may do something completely different to solve the same problem. Making general statements about the utility of "filter-based" pipelines doesn’t make really sense. We are only evaluating the utility of your "filter-based" pipeline. You should just use the name of the tool (Linear) when referring to it.

**Response:** Thank you for reviewing and commenting on the manuscript. We have read the comments of the first reviewer carefully. Our responses to the potential issues are as follows:

1. For the second item that the training and test data may overlap and thus may lead to issues such as data contamination and overfitting, we would like to clarify that the AAE has been appropriately settled in the work. First, from the perspective of data preparation, the training and test datasets are separated. Datasets for training and assessment in this work are from different sources shown in the following Table
   1. The training dataset is composed of a read dataset, which is based on filtered GIAB HG002 real reads, and a simulated SV dataset. The read dataset is prepared by first aligning HG002 reads and then applying additional filtration to aligned reads. Specifically, reads aligned to centromeres or containing indels *>* 100*bps* or aligned length *<* 0*.*8*×* read length or *<* 500*bps* are filtered out. There are about 5*.*3 million, 18% reads of HG002 selected for training. SVs are simulated with random lengths, and types and are planted into the prepared read datasets at random positions. Hence training and assessment datasets are largely separated.

Second, though deep learning can be powerful, AAE in this work is limited to the preliminary screening of discordant anchors to help initiate the generative model, which is free of training. Intuitively, the

idea of using AAE is to generate an overall impression of whether the anchors are likely from SVs and then tell the generative model whether to use parameters for assembling SVs by passing a continuous variable, the prior. Then the generative model uses dynamic programming (DP) to traverse and compute the likelihood of each potential assembly of sequence and choose the optimum one (i.e. of the maximum likelihood) as the result for output. Thus, the results are determined by the generative model. Without AAE, we can discretely initiate the generative model to consider gaps *>* 50*bps* for instance as a potential SV gap and use corresponding parameters for assembling. Many SV callers may employ such a method for screening gaps. The downside of such a screening is it takes explicitly defined discrete thresholds, which commonly are also arbitrary to some extent. For instance, a gap of 49*bps* would be filtered out by the above screening though it essentially has no significant difference in the chance of being an SV as 50*bps*. In contrast, AAE will tell the generative model that a gap of 50*bps* is probably an SV-related gap with prior=0.6. Though AAE tells the prior, it does not tell the final results, namely how the anchors are assembled, where the exact endpoints of the SVs are, etc. They will be resolved by the generative model.

Finally, from the perspective of results, the AAE results are empirical and inaccurate priors rather than ground truth, while the final results are accurate mappings that can be resolved by SAM/BAM-based applications. Thus the influence of the AAE on the final results is limited. It does not influence the algorithm of the generative model, which guarantees the results are independent of the AAE training dataset.

2. For the fourth item the filter-based pipeline in the work should have a distinct description and name instead of the general one, we have named it Leaf (LinEAr Filter) since it is implemented within Linear, which is a toolkit we developed for long-read analysis. Linear is the name of the toolkit, which may have other tools integrated in the future, thus we use Leaf as the name of the filter. We have replaced most occurrences of filter in the manuscript with Leaf, especially when it refers to a specific pipeline, such as Leaf-cuteSV or Leaf-SVIM. We also keep the filter in a few places, where we need to emphasize that it is a new type of pipeline as opposed to the aligner-based or assembly-based pipelines.

Table 1: Training and assessment (items 2-4) datasets in this work.

| Item | Read dataset | SV dataset |
| --- | --- | --- |
| 1. AAE training | HG002 Ashkenazim, filtered | Randomly simulated and planted intra-read SVs |
| 2. Trio-based | HG002-HG004 Ashkenazim | Real high-quality intra- and inter-read SVs |
|  | HG005-HG007 Han Chinese | Real high-quality intra- and inter-read SVs |
|  | SKBR3 cell line | Real nested SVs |
| 3. SV space | Systematically simulated | Systematically simulated, randomly planted intra-read |
|  |  | SVs |
| 4. Assembly-based | HG00733 PacBio HiFi | Real insertions and deletions called by dipcall |

## 1.2 New items of notes on the added assembly analysis:

-"However, a proportion of false positives are likely to be true positive SVs but are missed by the dipcall. The real recall and precision of the Filter-based pipelines are actually higher than the evaluated ones."

-This sounds like speculation to me. I’m sure there are some missed true positives, but you need to connect the dots between these statements and the quantification of the drop in precision and recall. You do mention the idea that dipcall could be biased towards minimap2-based benchmarks, and present using an NGMLR-based pipeline as a control for this bias, but once again, there needs to be a more quantified analysis for me to take those statements as fact.

-Also, it seems to me like there is a pretty unfavorable trade-off as you increase the evidence threshold. The alignment-based methods have pretty decent precision/recall with low evidence coverage. The filter method, on the other hand, starts with atrocious precision, and rises to the level of "OK" (at least for SVIM) as you

add evidence. But the recall drops to unacceptable levels before precision even approaches something decent. Please let me know if I am misinterpreting the precision/recall numbers here or if I am missing a key piece of information that helps put this observation into perspective.

**Response:** The dipcall (<https://github.com/lh3/dipcall>) assembly-based assessment is a special assess- ment that uses insertions and deletions detected by minimap2-based pipeline as the true SVs. The assess- ment workflow is as shown in the following Figure 1, where HPRC assembly is assembled by hifiasm [1] (<https://github.com/chhylp123/hifiasm>) developed by the minimap2 team. dipcall uses minimap2 to align HPRC assembly (<https://github.com/human-pangenomics/HPP_Year1_Assemblies/tree/main>) to the ref- erence genomes and call SVs by comparing the assembly-genome alignment. Both true dataset and test datasets are HG00733 PacBio HiFi reads. In such case, the assessment essentially uses minimap2 as the benchmark for evaluating the precision and recall of pipeline *X*, which is

*{SV s by X} ∩ {SV s by minimap*2 *for assembly* (*dipcall*)*}*

*recall_X_ ≈*

*SV s by minimap*2 *for assembly* (*dipcall*)

*recall_X_* is biased when *X* is minimap2-SV callers since the two sets in the expression largely overlap. Hence minimap2 in the test shown in manuscript Figure 6 is essentially employed as the reference of recall and precision bounds, while NGMLR-based pipeline is employed as the bias (confounder) control.

##### HG00733 HiFi read

*hifiasm*

HPRC assembly	*Leaf-SV caller*

###### dipcall minimap2-SV caller

##### True SVs Detected SVs

*Truvari*

##### recall and precision _5_

Figure 1: Datasets and tools used in the assembly-based assessment. Tools are marked with italic text. hifiasm and dipcall are minimap2-based. Truvari is a benchmark tool to compare SVs for recall and precision computation.

In such case, relative *recall_Leaf|minimap_*_2_ [2] defined in manuscript expression 1 is a more reasonable metric for recall assessment. The following table 2 shows the relative *recall_Leaf|minimap_*_2_, which suggests Leaf can recall most insertions and deletions detected by minimap2. It is worth noting that few tools can recall all

SVs (relative recall=1) detected by another tool. Thus the results suggest that Leaf achieves quite high recall in the assessment. On the other hand, the relatively lower precision of Leaf-based pipelines is attributable to that Leaf reported more SVs, some of which are regarded as false positives in the assessment. However, it conforms to the general design principle of filters, where sensitivity commonly takes priority over others including precision, which can be easily improved in the validation stage. Moreover, a number of the false positives are potential true SVs missed by dipcall. Research SVIM-asm [3] evaluated dipcall and suggests this number could be upper to 15% as shown in the following Figure 2, where recall of dipcall in the second row is appropriately 85%. Namely the true SV dataset can potentially miss 15% true SVs, which can largely contribute to the overestimated false positives leading to underestimated precision. Thus the real recall and precision of Leaf is substantially higher.

Finally, it is also worth noting that the assembly-based assessment only evaluated insertions and deletions, while one of the benefits of Leaf is detecting nonlinear intra-read SVs (i.e. inversions, duplications and nested

ones) missed by aligners such as shown in the inversion and duplication in the manuscript Figure 5 of the second assessment, where aligners are significantly ineffective in detecting inversions *<* 500*bps* for instance. These SVs are difficult for aligners in terms of algorithm level due to lacking effective gap models.

Table 2: *recallLeaf−cuteSV |minimap*2*−cuteSV* and *recallLeaf−SV IM|minimap*2*−SV IM* regarding the number of supporting reads (2-16) in the assembly-based assessment.

|  | | 2 | 4 | 6 | 8 | 10 | 12 | 14 | 16 |
| --- | --- | --- | --- | --- | --- | --- | --- | --- | --- |
| cuteSV | Leaf | 0.990861 | 0.968392 | 0.955160 | 0.943326 | 0.931093 | 0.917259 | 0.905167 | 0.888142 |
|  | NGMLR | 0.759630 | 0.758670 | 0.754535 | 0.744572 | 0.743849 | 0.734536 | 0.716043 | 0.694339 |
| SVIM | Leaf | 0.998574 | 0.948770 | 0.913454 | 0.889538 | 0.868488 | 0.846652 | 0.837556 | 0.819277 |
|  | NGMLR | 0.727796 | 0.726614 | 0.708845 | 0.697275 | 0.688478 | 0.685684 | 0.683353 | 0.674084 |

Figure 2: Dipcall SVIM-asm recall and precision reprinted from SVIM-asm research

## 1.3 New items of notes on the computational time analysis

–With the above notes in mind, it seems like the main advantage of the Filter-based pipeline presented is to provide a preprocessing step that could drastically speed up the SV calling process without hurting preci- sion/recall too much.

–You perform benchmarks using x1 up to x16 threads. From this benchmark, we begin to see diminishing returns from the Filter pipeline, but the benefits are still present at x16 threads.

–Here is my problem with this array of benchmarks. Modern computing clusters will have far more resources available than just x16 threads. I would like to see the analysis extended to x64 or even x128 threads.

–Will the main advantage of the Filter-based pipeline disappear?

–While it is true that the memory usage will increase it will still be far less than what many computing clusters have at their disposal. My laptop is on par with the current benchmarks.

**Response:** Regarding the computational benefits of multi-thread running:

1. In Figure 7 for runtime comparison in the original manuscript, the vertical axes are log-scaled while the horizontal axes are not, which may make lines look like diminishing more quickly than expected. In the

revised manuscript, we evaluated the elapsed time as well as the CPU time, which is a better metric for evaluating algorithm complexity, (i.e. CPU resource demands), as shown in Figure 7, where both vertical and horizontal axes are in log-scaled, to make the line trends clearer for comparison. It is worth noting that Leaf is upper to over 10 times faster than minimap2, and 50 times faster than NGMLR.

2. It is also worth noting that the elapsed time scaling nonlinearly for a growing number of threads are due to the limitation of Amdahl’s law. Namely, there exists the speed upper bound that using more threads will not be very productive for any of these tools, very few programs can scale well (linearly) with a growing number of threads. In the assessment, it is the input/output (I/O) latency that limits the speed upper bound. Since I/O commonly can not be parallelized, reading and writing large sizes sequences and result files involve intensive disk I/O, which will gradually become the computational bottleneck for a growing number of threads. Namely, parallelized threads for computation have to wait for the I/O. For a given number of threads *n*, the elapsed time and CPU time as mentioned above of running Leaf or aligners satisfy

*t*	⩾ max( *tCPU , t*	)

*Elapsed*

*n	I/O*

Leaf will reach the lower bound (i.e. *t_I/O_*) of elapsed time more quickly for a growing number of *n* as it has smaller *t_CP_ _U_* . The speed upper bound can be further increased if the hardware has lower I/O latency.

3. Finally, Leaf is designed to be a lightweight tool with a balanced performance of runtime and memory footprint in our original strategy. It takes moderate memory rather than rapidly increased memory consumption for a growing number of threads, and thus it can run on desktops and laptops. To this end, we limited the performance for multi-thread running. We have updated the source code to ease the limitation for running on clusters with, *×*64 for instance, threads. The update is available in the dev_io branch (not main branch yet) of the source code repository at <https://github.com/xp3i4/linear> and will be later merged to the main branch.

## 1.4 Conclusion

In my mind, the trade-off between loss of precision/recall on a high-quality assembly-based benchmark and the vanishing computational benefits with modern hardware leads me to believe that this method may not be as useful as initially thought. I still think that using a filter for reads before alignment/SV calling is an interesting idea, but as you mentioned in the discussion – perhaps the benefits of the filter can be maximized if an SV caller is tuned specifically for the filter or a new SV caller is built around the filter itself.

**Response:** The trade-off between loss of precision/recall compared to aligner-based pipelines mainly happens for insertion and deletion detection. Commonly, long-read aligners are capable of handling insertions and deletions by employing nonlinear models (e.g. convex model), though it is at the cost of largely increased computational resource demands. However, insertions and deletions are relatively simple rearrangements while aligners remain less effective in detecting more complex intra-read SVs, especially nonlinear (e.g. inverted, duplicated and nested) ones. The second assessment shown in Radar Figure 5 in the manuscript is also in line with that. The underlying cause is the limitation of the alignment algorithm complexity, which limits the capability of thoroughly taking into account potential rearrangements. The limitation has an insignificant impact on short-read SV detection pipelines since most short-read SVs are inter-read SVs supposed to be detected by SV callers. However, due to the ultra-long lengths, long reads commonly contain a significantly larger number of intra-read SVs, which can hardly be detected by SV callers if the SV signals have been lost by aligners, such as forced alignment. Therefore, a lightweight approach that can capture SV signals at an early stage would be helpful for long-read SV detection. To this end, we propose the filter-based pipeline to better resolve long-read SVs. As a conclusion for the features and potential benefits of Leaf in this work:

1. Leaf has comparable performance for long-read insertion and deletion detection.
2. Leaf significantly enhances the capability of aligners in detecting nonlinear intra-read SVs.
3. Leaf’s algorithm is essentially much more computationally efficient than long-read alignment.
4. It is also a technical validation revealing the feasibility and potential of filter-based pipelines for long-read

applications. The performance of filter-based pipelines would be further improved as a growing number of optimizations are employed.

# References

[1] Cheng, H., *et al.*: Haplotype-resolved de novo assembly using phased assembly graphs with hifiasm. Nature Methods **18**(2), 170–175 (2021). doi:10.1038/s41592-020-01056-5. Number: 2 Publisher: Nature Publishing

Group. Accessed 2022-10-18

[2] Frické,	M.:	Measuring	recall.	Journal	of	Information	Science	**24**(6),	409–417	(1998). doi:10.1177/016555159802400604. Publisher: SAGE Publications Ltd. Accessed 2024-02-02
[3] Heller, D., *et al.*: SVIM-asm: structural variant detection from haploid and diploid genome assemblies. Bioinformatics **36**(22-23), 5519–5521 (2021). doi:10.1093/bioinformatics/btaa1034. Accessed 2024-01-21

**Third round of review**

**Reviewer 1**

The authors have address all of my issues.
